# Enhanced anti-tumor immunotherapy by dissolving microneedle patch loaded ovalbumin

**DOI:** 10.1371/journal.pone.0220382

**Published:** 2019-08-06

**Authors:** Sung-Ju Lee, Hyeon-Seong Lee, Yun-Ho Hwang, Jong-Jin Kim, Kyung-Yun Kang, Seong Jin Kim, Hong Kee Kim, Jung Dong Kim, Do Hyeon Jeong, Man-Jeong Paik, Sung-Tae Yee

**Affiliations:** 1 Department of Pharmacy, Sunchon National University, Suncheon, Republic of Korea; 2 College of Pharmacy and Research Institute of Life and Pharmaceutical Sciences, Sunchon National University, Suncheon, Republic of Korea; 3 Center for Self-assembly and Complexity, Institute for Basic Science (IBS), Pohang, Republic of Korea; 4 Suncheon Research Center for Natural Medicines, Suncheon, Republic of Korea; 5 Research Center, RAPHAS Co., Ltd. #512 ABMRC, Yonsei University, College of Medicine, Seodaemun-gu, Seoul, Republic of Korea; Seoul National University College of Pharmacy, REPUBLIC OF KOREA

## Abstract

The skin is a very suitable organ for the induction of immune responses to vaccine antigens. Antigen delivery systems to the skin by needle and syringe directly deposit the antigen into the epidermal-dermal compartment, one of the most immunocompetent sites due to the presence of professional antigen-presenting cells aimed at the induction of antigen-specific T cells. In this study, we analyzed the amount of ovalbumin as an antigen delivered to the skin by a microneedle. When ovalbumin protein as an antigen was delivered to the skin of mice using a dissolving microneedle, it induced an immune response through the enhanced proliferation and cytokines production by the splenocytes and lymph nodes. Also, it effectively increased the ovalbumin-specific CD8^+^ T cell and CD4^+^ T cell population and induced an ovalbumin-specific CTL response against the graft of ovalbumin-expressing EG7 tumor cells in the immunized mice. Also, we identified the inhibition of tumor growth and prevention of tumor formation in the context of the therapeutic and prophylactic vaccine, respectively through EG-7 tumor mouse model. Finally, these data show the potential of patches as attractive antigen delivery vehicles.

## Introduction

Skin can induce an immune response in reaction to a small amount of antigen and consequently, is known as an effective target site for vaccine delivery by antigens [1–4]. The skin acts as the first immunological defense barrier [1–4]. Antigen-presenting cells, such as epidermal Langerhans cells and dermal dendritic cells, recognize antigens, and foreign agents coming from the corneum [1–4]. These cells readily uptake foreign antigens, migrate to the draining lymph node to present the antigen fragments to resting T lymphocytes, and initiate antigen-specific immune responses [1–4]. Vaccination through the skin is being studied and developed as an effective and successful approach to prevent infectious diseases and deliver therapeutic vaccines and anti-tumor vaccines [5–8]. Also, in the USA, vaccination has been recommended for decades to decrease the incidence of various diseases [9,10]. Vaccination has been reported to prevent almost 6 million deaths worldwide annually [9,10]. However, despite its various useful benefits it has recently been found to have several disadvantages [5,10–12]. Subcutaneous or intramuscular vaccination requires medical personnel, causes pain, fear and stress in children and their parents [5,10–12], and does not always induce an adequate specific immune response [5,13]. Therefore, recently, the use of a microneedle patch for the delivery of vaccine antigens has been studied in an attempt to overcome these disadvantages [5,10–12].

Antigen delivery systems aimed at the induction and enhancement of antigen-specific T cells through mature dendritic cells offer a promising approach to immunotherapy, due to their increased cellular immune response against the target [14,15]. Dendritic cells (DCs) are professional antigen-presenting cells (APCs) involved in immune responses that activate helper T cells and cytotoxic T cells (CD4^+^ T cells and CD8^+^ T cells) by antigen-cross presentation [16–25]. Recently, several studies demonstrated the important role of T cells in the fight against tumor [16]. CD8^+^ T cells play a central role in the host response to viral infections and cancer by inducing adaptive cellular immunity [17–22]. Also, CD8^+^ T cells have been shown to be a potent mediator of anti-tumor immunity, and tumor-directed immune-based therapies have focused on eliciting a cytotoxic T cell (CTL) response, primarily because CTLs can directly kill tumors and target cells [17–22]. Also, the induction of a Th1-type immune response by CD4^+^ T cell is essential for effective immunotherapeutic strategies [23–25]. Therefore, DCs induce cell-mediated immune responses and have anti-tumor effects on cytotoxic T cells [23–25]. Furthermore, the effects of DC-based anti-tumor vaccines are mediated by specialized interactions between DCs and T cells [23–25]. The microneedle patch has been shown to be an effective delivery technology, allowing for efficient vaccination and facile antigen delivery to the skin [1,26]. Immunization based on the antigen ovalbumin (OVA) model was studied using a novel transcutaneous delivery system consisting of an antigen-coated microneedle patch [1,5,27].

In this study, we investigate the applicability of the dissolving-microneedle-patch loaded antigen for vaccination purposes by using OVA as a model antigen for the induction of an immune response [1,5,27]. First of all, we investigated whether transdermal delivery systems using the dissolving-microneedle-patch loaded OVA induced or enhanced a cellular immune response against the target of interest based on antigen-specificity. Also, we demonstrated that the antigen-specific immune response from the subunit antigens delivered via the patch induced anti-tumor activity by significantly enhancing the Th1 cell and cytotoxic T cell response.

## Materials and methods

### Dissolving microneedle fabrication via DAB

All of the dissolving microneedle patches used in this study were fabricated by the “Droplet-born air blowing (DAB)” method. In this study, the fabrication process followed that described in a previous paper [11]. Briefly, a viscous solution of hyaluronic acid (HA) and OVA was prepared by means of a planetary centrifugal vacuum mixer. Precisely controlled droplets were placed on a hydro-colloid sheet by a solution dispenser (control patch-HA, OVA patch-HA+OVA). After that, the dispensed viscous droplets on the lower sheet came into contact with the upper sheet and became elongated. To solidify the elongated viscous materials, the air was blown on them for a sufficient time at room temperature. Finally, a separation step was performed. These steps were repeated for the production of each dissolving microneedle patch via DAB.

### Animals and experimental treatments in vivo

Female 8- to 12-week-old C57BL/6 mice, weighing 20–22 g each, were purchased from Orientbio (Orientbio Inc., Seongnam, Korea). The animals were housed in a controlled environment [22 ± 2 and 50 ± 5% (relative humidity)] in polycarbonate cages and fed a standard animal diet with water. For the in vivo experiments, the mice were randomly divided into two groups of 4 animals each. The control group mice were treated by microneedle at the ventral site, and the treatment group mice were subjected to ovalbumin delivery by microneedle at the ventral site. All of the mice were treated in strict accordance with the guidelines issued for the care and use of laboratory animals by the Sunchon National University Institutional Animal Care and Use Committee (SCNU IACUC). All procedures were approved by the SCNU IACUC (Permit Number: SCNU IACUC-2016-12)

### Reagents and antibodies

OVA and trifluoroacetic acid (TFA) were purchased from Sigma-Aldrich (Steinheim, Germany). HPLC grade water and acetonitrile were purchased from Daejung Chemical (Siheung-si, Gyeonggi-do, Republic of Korea). Spin-X centrifuge filter 0.45 μm with cellulose acetate was purchased from Costar (Corning Incorporated, Corning, NY, USA). The following FITC- or PE-conjugated monoclonal anti bodies (Abs) and non-labeled Abs were purchased from BD Bioscience (San Joes, CA, USA): FITC- IFN-γ, PE-CD8, and CD16/32 (2.4G2). The cytokine ELISA primary and secondary–antibodies specific for murine IL-2, IFN-γ were purchased from BD Biosciences (San Jose, CA, USA). The 5-Bromo-2 –Deoxy-Uridine Labeling and Detection Kit III, and collagenase D was purchased from Roche (Salt Lake City, UT, USA).

### High performance liquid chromatography analysis

**Fig 1:**
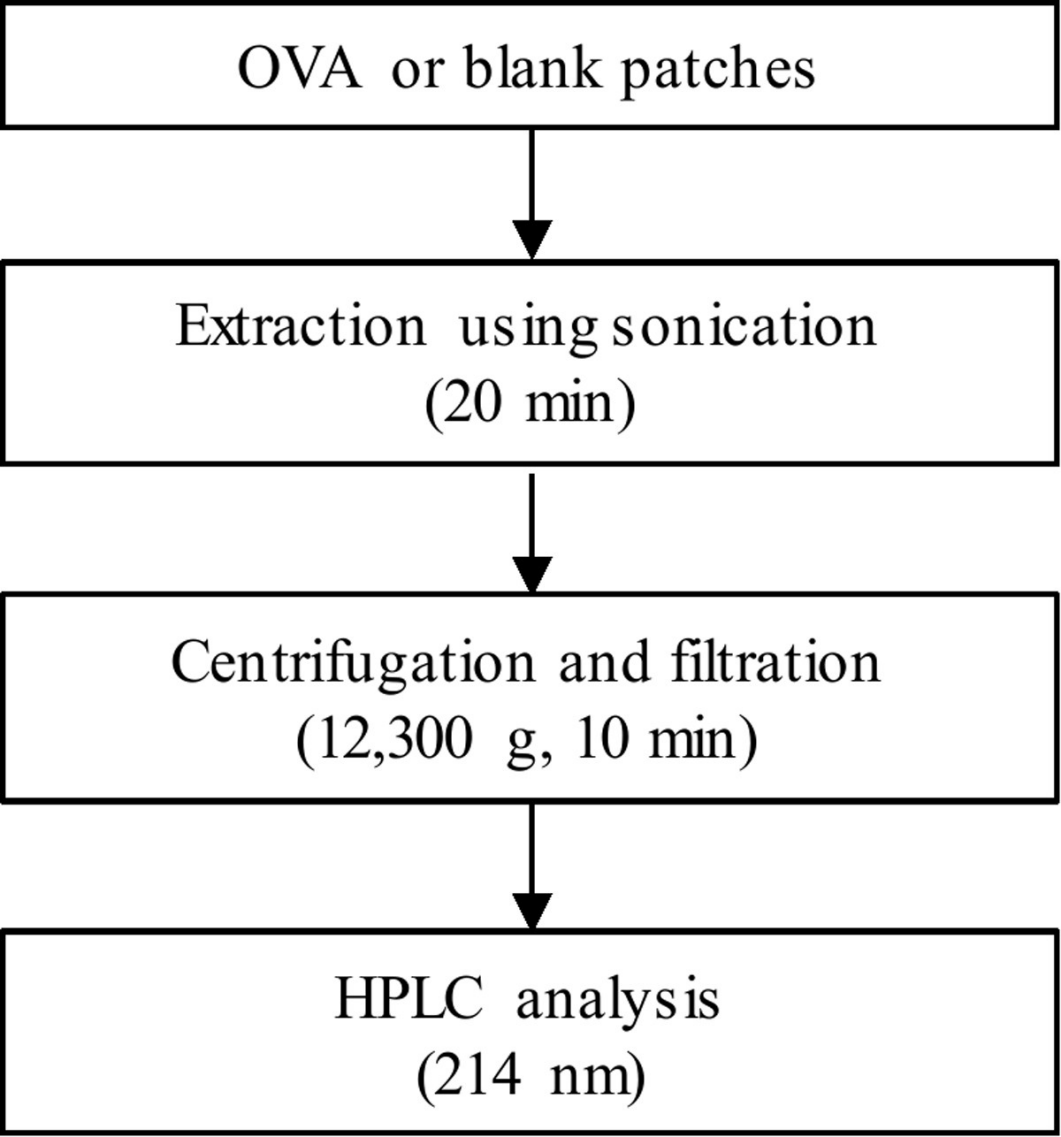
Experimental scheme of OVA tor HPLC analysis. DOI: 10.6084/m9.figshare.7730387.

High performance liquid chromatography (HPLC) analysis was performed with a Shimadzu Prominence HPLC system (Shimadzu Corp., Kyoto, Japan) coupled with a SPD-M20A Photodiode Array Detector (Shimadzu Corp., Kyoto, Japan). Chromatographic separation was performed using Jupiter C4 column (5 μm, 300 Å, 150 × 4.6 mm id, Phenomenex) proceeded by a 20 × 4.6 mm guard column. The column oven was maintained at 40°C and the auto-sampler temperature at 4°C. Mobile phase A (water, 0.025% TFA) and mobile phase B (acetonitrile, 0.025% TFA) was used as solvent system. Gradient elution of mobile phase B was initiated from 15% (1 min), increased from 15% to 100% (8 min), and then held at 100% for 11 min, total flow rate was 1.0 mL/min. Ultraviolet (UV) absorption spectra were monitored at 214 nm. Experimental scheme of OVA tor HPLC analysis is Fig 1.

### Sample preparation for OVA analysis in dissolving-microneedle-patch loaded OVA

The present method for OVA analysis was performed as the procedure by Grotefend, S., et al. (2012) [28]. Standard solution of the OVA made up at 1.0 mg/mL in water with 0.025% TFA. Dissolving-microneedle-patch loaded OVA (OVA patch) and non-loaded OVA (blank patch) were dissolved in 1.0 mL water with 0.025% TFA under a sonication for 20 min. Patch extracts (300 μL) was centrifuged at 12,300 g for 10 min after transferring to the Spin-X centrifuge filter. Aliquot (100.0 μL) of filtered extracts was injected into the HPLC system for analysis.

### Cell culture

E.G7-OVA, a chicken egg OVA gene-transfected clone of EL4, which presents OVA with MHC class I molecules, were obtained from the American Type Culture Collection (Manassas, VA, USA) [29].

### In vivo efficacy study of dissolving-microneedle-patch loaded OVA

The skin of the ventral region was shaved, and 10μg of OVA was delivered into the skin at the ventral site of the mice using an OVA dissolving microneedle patch. These microneedle patches were removed from the mice for 3 hours once a week for three weeks. One week after delivering the last microneedle patches, the harvested spleens and axillary lymph nodes, brachial lymph nodes, and inguinal lymph nodes were dispersed by tweezers, pooled, and treated with red blood cell lysis buffer.

### In vitro stimulation of mouse splenocytes and lymph nodes

The splenocyte and lymphocyte cells obtained from the spleen and lymph nodes of the two groups of C57BL/6 mice, respectively, were cultured in RPMI medium and then re-stimulated with OVA at concentrations of 50 μg/ml, 100 μg/ml, and 1000 μg/ml or not re-stimulated. After 24 h of incubation, we measured the cytokine production with the ELISA assay. After 48 h of incubation, the spleen cell and lymph node cell proliferation were determined using a 5-bromo-2-deoxy-uridine labeling and detection kit III. The results are expressed as the mean of triplicate experiments. The intracellular cytokine concentrations were determined by flow cytometry to analyze the cytotoxic T cell population.

### Cytokine assay

The culture supernatants were analyzed by the enzyme-linked-immunosorbent assay (ELISA). The levels of the various cytokines secreted by the splenocytes and lymphocyte cells re-stimulated with OVA were measured by ELISA.

### Intracellular cytokine staining

The CD4^+^ T cells, and CD8^+^ T cells were treated with in the presence of anti-FcR (2.4G2), fixed with 4% paraformaldehyde in PBS, permeabilized with 0.1% saponin, and stained with FITC-anti-IFN-γ and PE-anti-CD8. The CD8^+^ T cells were then gated and analyzed using a FACScanto II (BD Biosciences).

### Cytotoxicity assay

One week after delivering the last microneedle patch which allows OVA to penetrate into the skin, splenocytes were used as the effector cells without in vitro stimulation, and EG7-OVA cells were used as the target cells. 1 × 10^4^ cells target cells were incubated with effector cells at effector/target ratio of 50/1 and 100/1 in 96-well round bottom plates. The CTL activity after 4h was measured with the lactate dehydrogenase (LDH) cytotoxicity detection assay and evaluated at various ratios of effector cells to target cells (EG7-OVA) using an EZ-LDH Cell Cytotoxicity Assay Kit (Dogen) according to the manufacturer’s instructions. The cytotoxicity (%) was calculated using the following equation: (Experimental values of Effector Cell Spontaneous Control-Target Cell Spontaneous Control)/(Target Cell Maximum Control-Target Cell Spontaneous Control) x 100 [30]. The secretion of IFN-γ cytokines caused by the cytotoxicity activity was determined by ELISA after 24h.

### Anti-tumor activity

To measure the therapeutic efficacy, the C57BL/6 mice were subjected to the subcutaneous injection of 1×10^5^ E.G7 cells, an OVA-expressing EL4 variant, into a flank site and, then, the dissolving-microneedle-patch loaded OVA was inserted into the skin at the ventral site of the mice on days 1, 8, and 15 after tumor inoculation. On day 22, the mice were euthanized. For the prophylaxis experiments, these dissolving-microneedle-patch were put on the skin at the ventral site of the mice for 3 hours once a week for three weeks. One week after delivering the last dissolving-microneedle-patch, the mice were subjected to a subcutaneous injection of 1 × 10^5^ E.G7 cells into a flank site. When the average tumor diameter was 1–2 cm, the mice were euthanized. Tumor growth was monitored and measured every 2 days (n = 4 mice/group) by measuring the major and minor axes of the tumors using calipers. The tumor volume was calculated using the following formula: tumor volume (mm3) = major axis×minor axis^2^×0.5.

### Statistical analysis

The results are presented as means±SD. Statistical analyses were performed using the SPSS program (SPSS, Chicago, IL, USA). Mann–Whitney U test and Kruskal-Wallis test were used for comparisons between two groups or within group; respectively. Probability values of < 0.05 were considered significant (P values are indicated as follows: (P values *<0.05, **<0.01, ***<0.001, ^#^<0.05, ^##^<0.01, ^###^<0.001)

## Results

### Analysis of antigen amount delivered to skin by dissolving-microneedle-patch loaded OVA (OVA analysis by HPLC)

**Fig 2:**
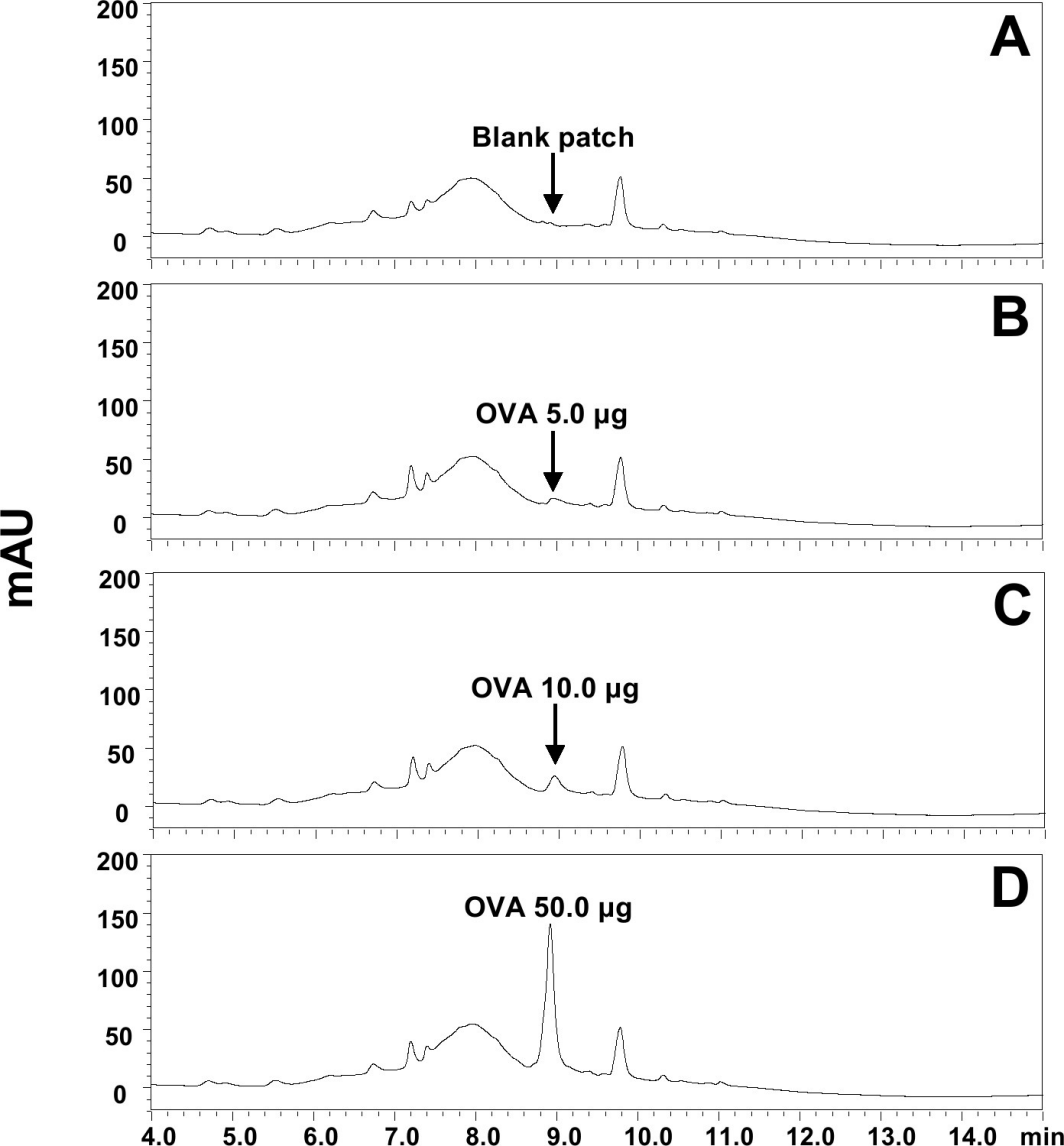
**Representative chromatograms of OVA in extracts of (A) blank patch, (B) blank patch + OVA 5.0 μg, (C) blank patch + OVA 10.0 μg, and (D) blank patch + OVA 50.0 μg**. Digital Object Identifier: 10.6084/m9.figshare.7679813.

**Fig 3:**
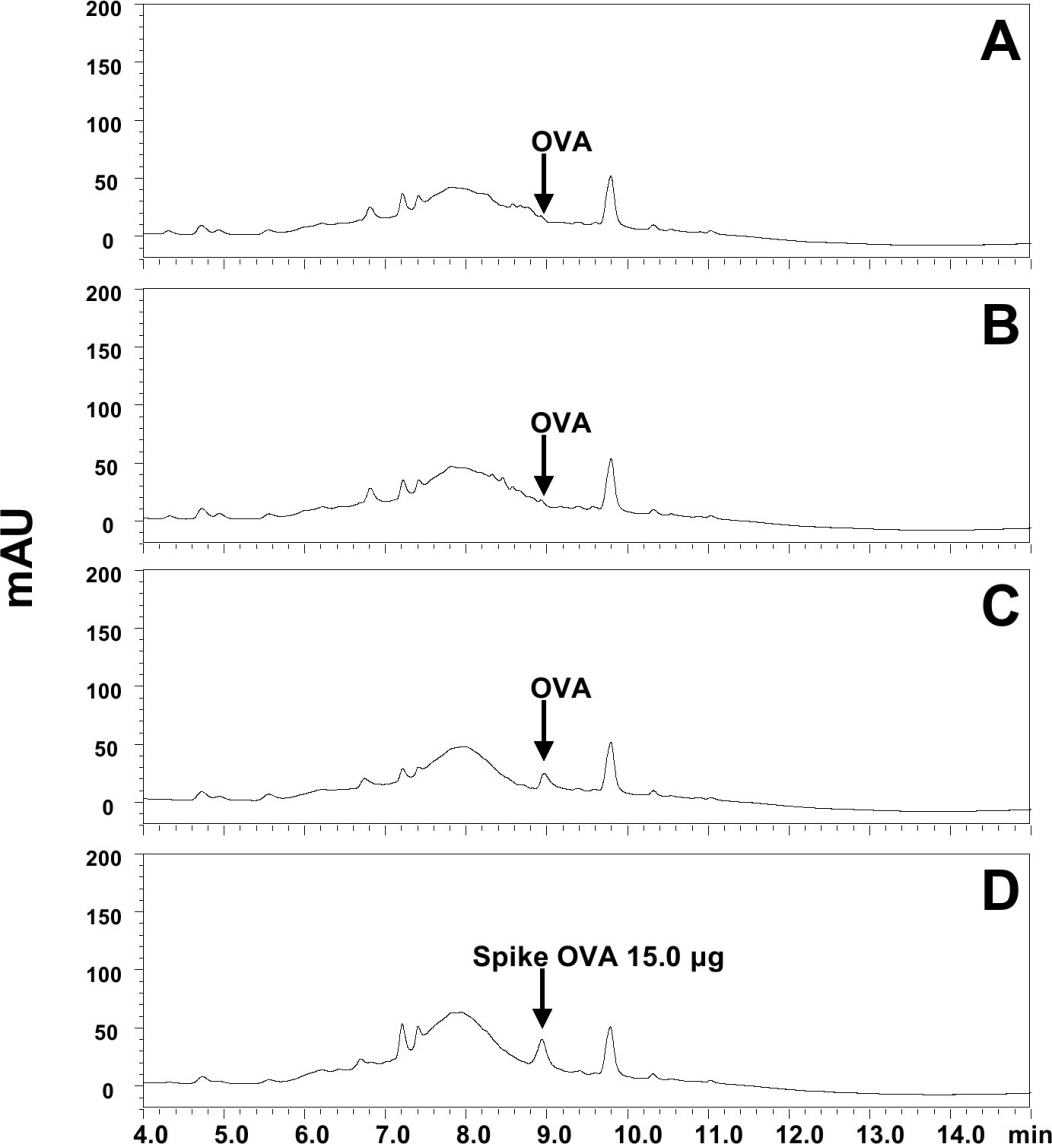
**Representative chromatograms of OVA in extracts of (A) blank patch and (B) OVA patch extracts after applying to the skin, (C) OVA patch before applying OVA patch to the skin, (D) OVA 15.0 μg + OVA patch extracts after applying to the skin.** Digital Object Identifier: 10.6084/m9.figshare.7679822.

The OVA was detected as a single peak at 8.9 min in extracts of a blank patch spiked with OVA standard. The chromatograms of OVA in extracts of blank patch (2A), and extracts of blank patches spiked with OVA of 5.0 (2B), 10.0 (2C), and 50.0 μg (2D) showed in Fig 2, which showed high peak height in proportion to the increase of OVA. In Fig 3, OVA peak was not detected in blank (3A) and OVA patch (3B) extracts after applying OVA patch to the skin. The chromatogram of Fig 3C showed OVA peak in commercial patch product before applying OVA patch to the skin, which was similar with OVA level in blank patch extracts with spiked OVA standard 10.0 μg (Fig 2C). And chromatogram of Fig 3D showed OVA peak in patch extracts after applying OVA patch to the skin spiked with OVA of 15 μg. Therefore, these may explain that most of OVA (or more than 99%) in patch was delivered into skin (or body). The values used to Figs 2 and 3 is Table 1.

**Table 1:** The peak areas of OVA in patch extracts and OVA standards.

Analytes	Peak area																			
	Blank patch extracts with spiked OVA standards			OVA patch extracts with spiked OVA standard^a^	before applying patch to the skin								after applying patch to the skin							
	5.0 μg	10.0 μg	50.0 μg	15.0 μg	Blank patches			Mean ± SD	OVA patches			Mean ± SD	Blank patches			Mean ± SD	OVA patches			Mean ± SD
Ovalbumin	37,779	146,021	1,029,026	194,795	4,382	6,430	5,054	5,289 ± 1,044	115,591	115,121	113,802	114,838 ± 927	5,269	5,225	4,095	4,863 ± 665	5,204	5,214	5,284	5,234 ± 44

^a^OVA 15.0μg +OVA patch extracts after applying to the skin: The values (peak area) is the response of absorbance at 214 nm for detection of OVA by HPLC analysis. These values (peak area) showed as mean+SD values.

### Effect of dissolving-microneedle-patch loaded OVA on splenocyte responses in mice

**Fig 4:**
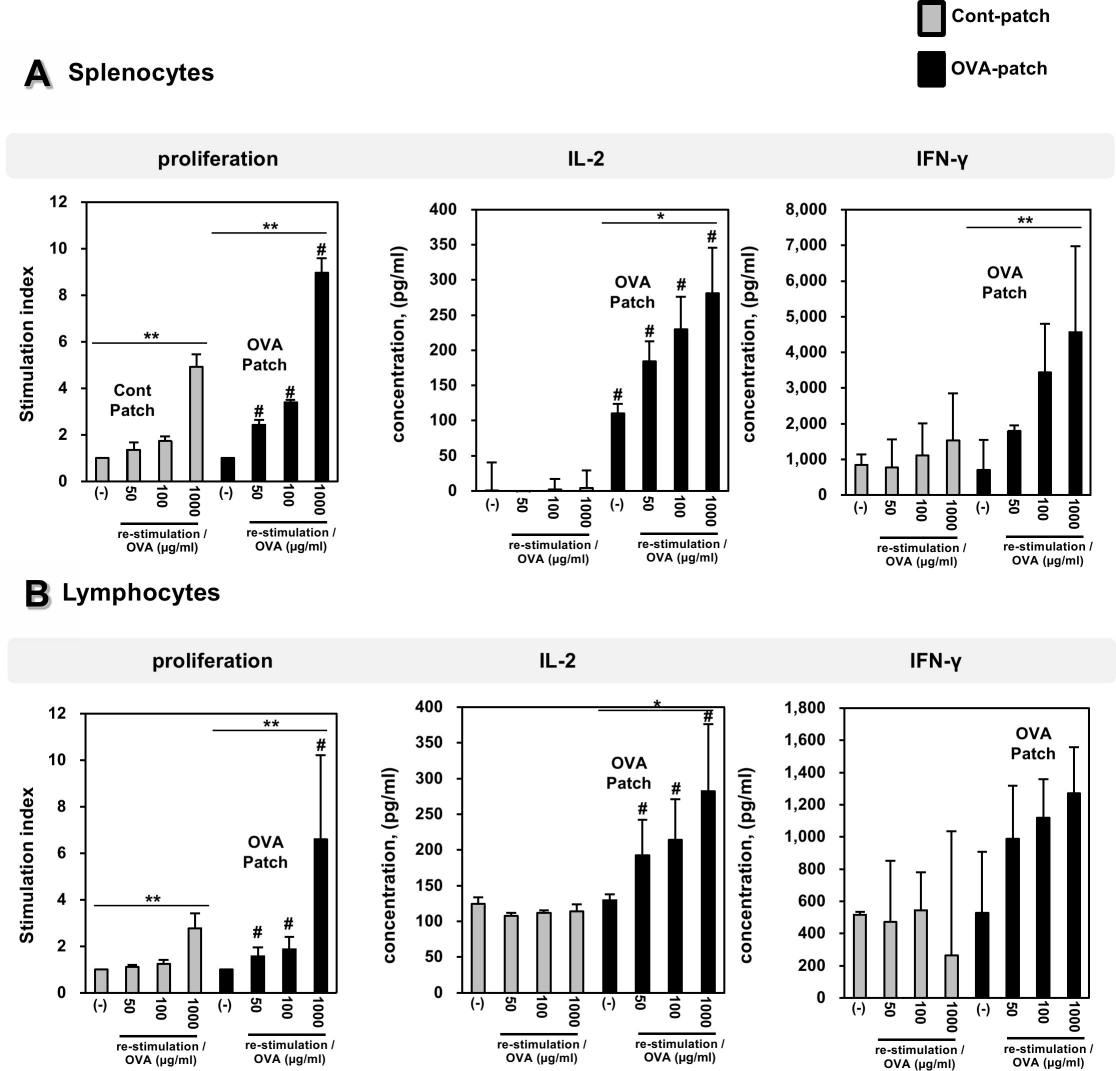
Effect of dissolving-microneedle-patch loaded OVA on splenocytes and lymphocytes proliferation in mice. The mice (n = 4/group) were immunized either using the dissolving-microneedle-patch or dissolving-microneedle-patch loaded OVA. The splenocytes and lymphocytes of each group were re-stimulated with OVA 7 days after the last immunization. The proliferation was assessed using a Bromo-kit. The culture supernatants were harvested after 24 h, and the cytokine levels were measured by ELISA. Data are represented as mean ± SD of four mice per group. *p<0.05, **p<0.01 or **p<0.001 (-) vs. re-stimulation OVA. Per group. ^#^p<0.05, ^##^ p<0.01 or ^###^ p<0.001 control-patch group vs. OVA-patch group. Digital Object Identifier: 10.6084/m9.figshare.7679675.

When the OVA protein as an antigen is delivered to the skin using the dissolving-microneedle-patch, the OVA should be released to induce an immune response. And we quantified the cellular expansion of antigen-specific to verity the in vivo vaccination activity of the dissolving-microneedle-patch loaded OVA. To determine the delivery of dissolving-microneedle-patch loaded OVA and to test the level of immunization against OVA obtained using the dissolving-microneedle-patch, the splenocytes and lymphocytes from each experimental mouse were subjected to OVA re-stimulation. To test the dependency of the level of immunization on the amount of dissolved antigen, OVA at concentrations of 50 μg/ml, 100 μg/ml, and 1000 μg/ml were re-stimulated. The effects of the dissolving-microneedle-patch loaded OVA on the splenocyte and lymphocytes proliferation responses to OVA re-stimulation are shown in Fig 4. After OVA re-stimulation, the splenocytes and lymphocytes from the mice immunized with OVA exhibited an enhanced cell proliferation response. Likewise, the amounts of IL-2 and IFN- γ present in the supernatants obtained from the mice immunized with OVA were greater than those of the mice in the control group (Fig 4B). These results suggest that the dissolving-microneedle-patch loaded OVA and delivered the OVA antigens and induced greater immunization. These cytokine profiles indicate that immunization by the dissolving-microneedle-patch loaded OVA produces a OVA-specific Th1 cell response. The values used to Fig 4 is Table 2.

**Table 2:** Effect of dissolving-microneedle-patch loaded OVA on splenocytes and lymphocytes proliferation and cytokine production in mice.

**Splenocytes Proliferation**									
		mice		1	2	3	4	mean	s.d
Stimulation Index	Cont-Patch	(-)		1.00	1.00	1.00	1.00	1.00	0.0
		re-stimulation OVA(μg/ml)	50	1.82	1.28	1.11	1.22	1.36	0.3
			100	2.02	1.63	1.60	1.67	1.73	0.2
			1000	5.59	5.12	4.51	4.44	4.92	0.5
	OVA-Patch	(-)		1.00	1.00	1.00	1.00	1.00	0.0
		re-stimulation OVA(μg/ml)	50	2.15	2.39	2.53	2.64	2.43	0.2
			100	3.30	3.34	3.51	3.43	3.39	0.1
			1000	8.13	8.83	9.41	9.49	8.97	0.6
**Splenocytes IL-2**									
		mice		1	2	3	4	mean	s.d
Cytokine (pg/ml)	Cont-Patch	(-)		-51.32	7.00	45.39	2.59	0.92	39.8
		re-stimulation OVA(μg/ml)	50	-64.39	-13.74	-20.84	-24.71	-30.92	22.8
			100	-9.87	-6.00	23.03	2.71	2.47	14.7
			1000	-31.16	4.16	20.13	23.84	4.24	25.1
	OVA-Patch	(-)		117.39	93.68	107.06	123.52	110.41	13.1
		re-stimulation OVA(μg/ml)	50	172.71	199.16	150.94	215.45	184.56	28.5
			100	271.90	169.97	216.58	261.10	229.89	46.6
			1000	321.74	304.65	184.00	312.87	280.81	64.9
**Splenocytes IFN-Ɣ**									
		Mice		1	2	3	4	mean	s.d
Cytokine (pg/ml)	Cont-Patch	(-)		584.00	637.75	1004.00	1181.50	851.81	288.3
		re-stimulation OVA(μg/ml)	50	-109.75	385.25	1170.25	1654.00	774.94	788.2
			100	-19.75	826.50	1561.50	2062.75	1107.75	907.1
			1000	-182.25	1225.25	2376.50	2724.00	1535.88	1312.3
	OVA-Patch	(-)		-547.25	1106.50	1141.50	1147.75	712.13	839.8
		re-stimulation OVA(μg/ml)	50	1620.25	1705.25	1912.75	1941.50	1794.94	157.0
			100	1732.75	2964.00	4349.00	4715.25	3440.25	1365.5
			1000	1997.75	3054.00	6314.00	6902.75	4567.13	2408.2
**Lymphocytes Proliferation**									
		Mice		1	2	3	4	mean	s.d
Stimulation Index	Cont-Patch	(-)		1.00	1.00	1.00	1.00	1.00	0.0
		re-stimulation OVA(μg/ml)	50	1.10	0.99	1.18	1.15	1.11	0.1
			100	1.18	1.02	1.34	1.40	1.24	0.2
			1000	2.70	1.90	3.07	3.41	2.77	0.6
	OVA-Patch	(-)		1.00	1.00	1.00	1.00	1.00	0.0
		re-stimulation OVA(μg/ml)	50	1.34	1.32	1.46	2.13	1.56	0.4
			100	1.50	1.52	1.82	2.63	1.87	0.5
			1000	4.34	4.22	5.95	11.88	6.60	3.6
**Lymphocytes IL-2**									
		mice		1	2	3	4	mean	s.d
Cytokine (pg/ml)	Cont-Patch	(-)		134.81	117.55	115.45	129.32	124.28	9.3
		re-stimulation OVA(μg/ml)	50	104.16	113.52	106.26	106.10	107.51	4.1
			100	112.23	111.74	108.19	115.77	111.98	3.1
			1000	102.87	109.81	125.29	117.71	113.92	9.7
	OVA-Patch	(-)		135.45	120.45	124.48	137.39	129.44	8.3
		re-stimulation OVA(μg/ml)	50	150.45	206.90	157.39	256.42	192.79	49.3
			100	169.32	261.74	159.97	264.81	213.96	57.1
			1000	337.55	225.77	182.39	383.68	282.35	94.0
**Lymphocytes IFN-Ɣ**									
		mice		1	2	3	4	mean	s.d
Cytokine (pg/ml)	Cont-Patch	(-)		580.25	631.50	795.25	817.75	706.19	118.1
		re-stimulation OVA(μg/ml)	50	495.25	532.75	591.50	691.50	577.75	85.6
			100	522.75	722.75	776.50	1156.50	794.63	264.8
			1000	374.00	662.75	997.75	1330.25	841.19	413.8
	OVA-Patch	(-)		390.25	784.00	974.00	1011.50	789.94	284.5
		re-stimulation OVA(μg/ml)	50	702.75	911.50	1541.50	1562.75	1179.63	438.6
			100	979.00	1111.50	1537.75	1851.50	1369.94	399.9
			1000	516.50	1204.00	1279.00	1916.50	1229.00	572.5

### Effect of dissolving-microneedle-patch loaded OVA on OVA-specific T cell response in mice

**Fig 5:**
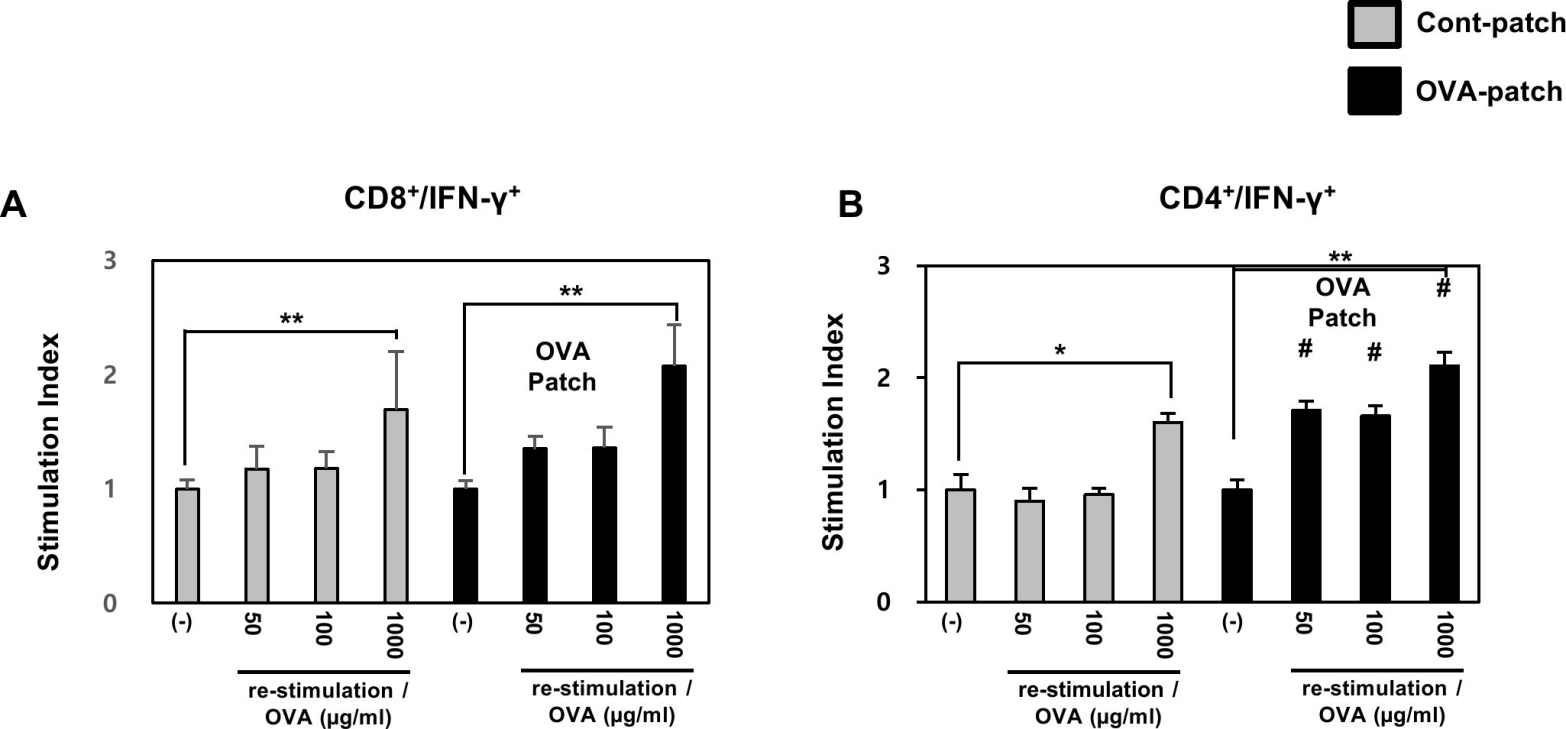
Effect of dissolving-microneedle-patch loaded OVA on OVA-specific T cell response in mice. The mice (n = 4/group) were immunized either using the dissolving-microneedle-patch or dissolving-microneedle-patch loaded OVA. The splenocytes of each group were re-stimulated with OVA 7 days after the last immunization and CD4^+^, and CD8^+^ T cells were then gated and analyzed using a FACScanto II (BD Biosciences) by staining with FITC-anti-IFN-γ and PE-anti-CD4/CD8. Data are represented as mean ± SD of four mice per group. *p<0.05, **p<0.01 or **p<0.001 (-) vs. re-stimulation OVA. per group. ^#^p<0.05, ^##^ p<0.01 or ^###^ p<0.001 control-patch group vs. OVA-patch group. Digital Object Identifier: 10.6084/m9.figshare.7726043.

IL-2 and IFN-γ are important markers of T cell survival and activation. To further demonstrate the antigen-specific activity of the dissolving-microneedle-patch loaded OVA in activating the antigen-specific T cells in vivo, we measured the secretion of intracellular cytokines in vitro. After the 3rd boost immunization, the endogenous OVA-specific T cell responses were evaluated by determining the upregulated expression of the activation marker, IFN-γ, on splenic CD4^+^ T cells and splenic CD8^+^ T cells by intracellular staining. Fig 5, shows that the percentage of OVA-specific CD4^+^ T cells/ -CD8^+^ T cells in the mice that received OVA was significantly higher than that in the control mice.

Furthermore, a trend was observed toward a higher percentage of OVA-specific CD4^+^ T cells/ -CD8^+^ T cells upon OVA delivery by dissolving-microneedle-patch loaded OVA compared to control microneedle patch. This result showed that the dissolving-microneedle-patch loaded OVA induced the OVA-specific T cell response by increasing the production mediated activation and proliferation of cytotoxic T cells and helper T cells. The values used to Fig 5 is Table 3.

**Table 3:** Effect of dissolving-microneedle-patch loaded OVA on OVA-specific CD4^+^/CD8^+^ T cell response in mice.

**CD8+/IFN-Ɣ+**									
		mice		1	2	3	4	mean	s.d
Percentage, %	Cont-Patch	(-)		5.80	10.30	11.40	13.95	10.36	3.4
		re-stimulation OVA(μg/ml)	50	7.40	10.50	15.60	14.40	11.98	3.7
			100	6.55	12.05	15.40	14.70	12.18	4.0
			1000	14.35	15.10	18.60	16.90	16.24	1.9
	OVA-Patch	(-)		10.10	9.10	9.35	23.60	13.04	7.1
		re-stimulation OVA(μg/ml)	50	14.85	12.55	11.75	30.70	17.46	8.9
			100	11.35	14.10	12.85	32.75	17.76	10.1
			1000	18.65	23.50	17.45	47.25	26.71	13.9
**CD8+/IFN-Ɣ+**									
		mice		1	2	3	4	mean	s.d
Stimulation Index	Cont-Patch	(-)		1.00	1.00	1.00	1.00	1.00	0.0
		re-stimulation OVA(μg/ml)	50	1.28	1.02	1.36	1.05	1.18	0.2
			100	1.13	1.17	1.35	1.07	1.18	0.1
			1000	2.47	1.47	1.63	1.22	1.70	0.5
	OVA-Patch	(-)		1.00	1.00	1.00	1.00	1.00	0.0
		re-stimulation OVA(μg/ml)	50	1.47	1.39	1.30	1.25	1.35	0.1
			100	1.12	1.56	1.39	1.38	1.36	0.2
			1000	1.85	2.61	2.00	1.86	2.08	0.4
**CD4+/IFN-Ɣ+**									
		mice		1	2	3	4	mean	s.d
Percentage, %	Cont-Patch	(-)		3.50	3.90	4.30	4.80	4.13	0.6
		re-stimulation OVA(μg/ml)	50	2.70	4.00	4.10	4.10	3.73	0.7
			100	3.60	3.80	3.90	4.40	3.93	0.4
			1000	5.90	6.40	6.80	7.20	6.58	0.7
	OVA-Patch	(-)		3.30	3.40	3.50	4.00	3.55	0.4
		re-stimulation OVA(μg/ml)	50	5.30	6.10	6.10	6.80	6.08	0.6
			100	5.60	5.80	6.00	6.10	5.88	0.2
			1000	7.10	7.50	7.50	7.70	7.45	0.3
**CD4+/IFN-Ɣ+**									
		mice		1	2	3	4	mean	s.d
Stimulation Index	Cont-Patch	(-)		0.85	0.95	1.04	1.16	1.00	0.1
		re-stimulation OVA(μg/ml)	50	0.77	1.03	0.95	0.85	0.90	0.1
			100	1.03	0.97	0.91	0.92	0.96	0.1
			1000	1.69	1.64	1.58	1.50	1.60	0.1
	OVA-Patch	(-)		0.93	0.96	0.99	1.13	1.00	0.1
		re-stimulation OVA(μg/ml)	50	1.61	1.79	1.74	1.70	1.71	0.1
			100	1.70	1.71	1.71	1.53	1.66	0.1
			1000	2.15	2.21	2.14	1.93	2.11	0.1

### Immunization by dissolving-microneedle-patch loaded OVA induces OVA-specific CTL activity

**Fig 6:**
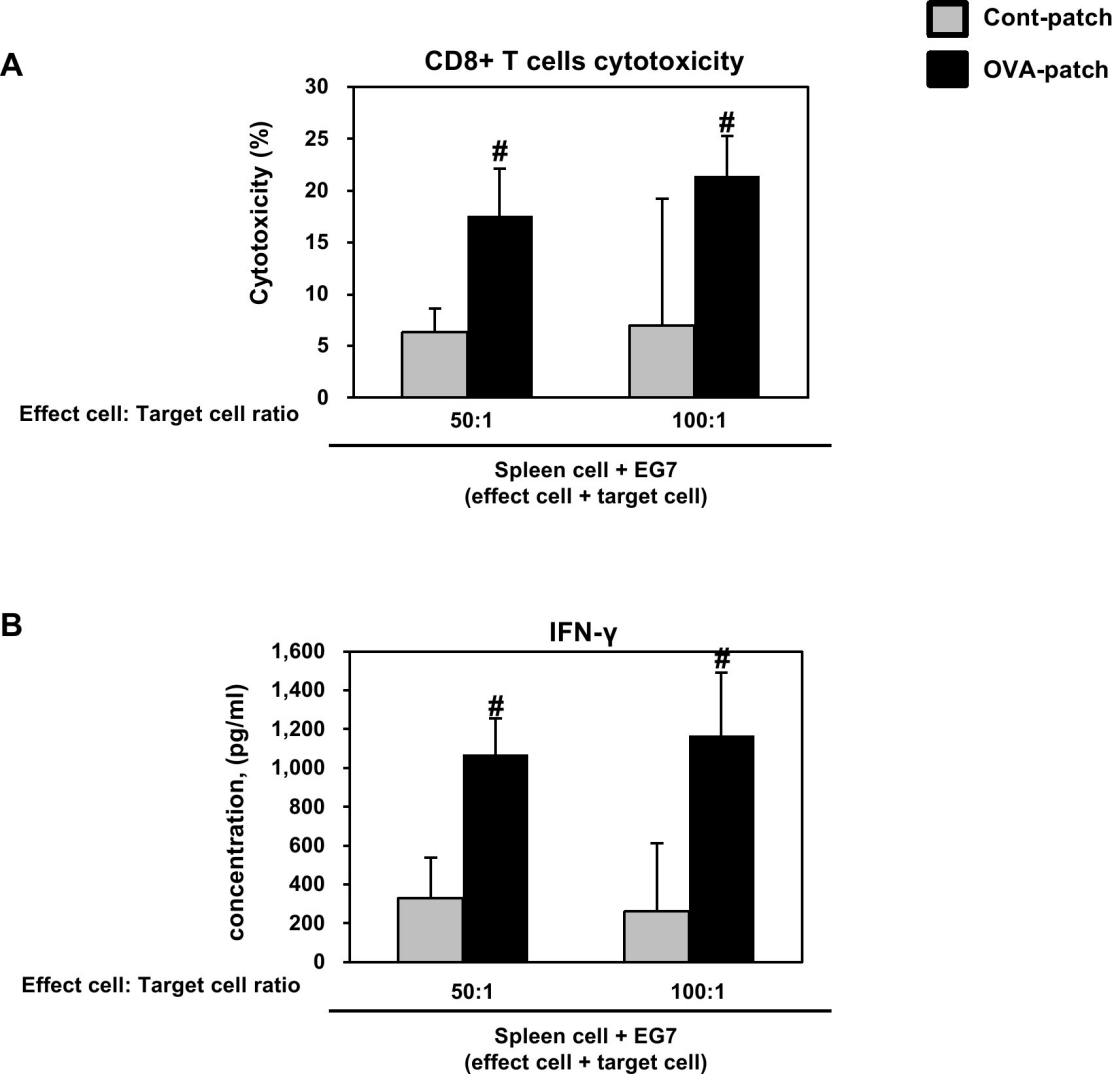
**(A) Immunization by dissolving-microneedle-patch loaded OVA induces OVA-specific CTL activity and (B) cytokine levels (IFN-ɣ).** The mice (n = 4/group) were immunized either using the dissolving-microneedle-patch or dissolving-microneedle-patch loaded OVA. 7 days after the last immunization, the mice were sacrificed and the splenocytes of each group were harvested for the in vitro killing assay (E/T = 50, and 100). Data are represented as mean ± SD of four mice per group. ^#^p<0.05, ^##^ p<0.01 or ^###^ p<0.001 control-patch group vs. OVA-patch group. Digital Object Identifier: 10.6084/m9.figshare.7679693.

Based on the observation that the dissolving-microneedle-patch loaded and delivered the OVA antigens and induced greater immunization as an adjuvant to stimulate OVA-specific CD8^+^ T cell activation, we further investigated whether this response could protect the mice from a graft of OVA-expressing EG7 tumor cells. The cytolytic activity of the responding T cells was tested directly on the OVA-expressing EG7 target cells in the LDH-release assay and the IFN-γ cytokine secretion through the cytotoxicity activity of the CD8^+^ cells. As shown in Fig 6, these results suggested that the dissolving-microneedle-patch loaded OVA induced an OVA-specific CTL response that targeted the EG7 tumor cells in the mice immunized with OVA. Collectively, these results indicate that when the microneedle coated with OVA protein as an antigen is delivered to the skin, the OVA is released, thereby inducing an immune response, and then the OVA-specific CTLs have the potential to kill the OVA-expressing tumor cells in vivo. The values used to Fig 6 is Table 4.

**Table 4:** Immunization by dissolving-microneedle-patch loaded OVA induces OVA-specific CTL activity and cytokine levels (IFN-ɣ).

**CD8+ T cell Cytotoxicity**									
		mice		1	2	3	4	mean	s.d
Cytotoxicity, %	Cont-Patch	E:T	50:1	8.59	3.58	5.26	7.87	6.33	2.3
			100:1	8.78	-10.8	16.45	13.48	6.98	12.3
	OVA-Patch	E:T	50:1	14.6	14.63	24.25	16.76	17.56	4.6
			100:1	23.69	25.41	19.76	16.95	21.45	3.8
**CD8+/IFN-Ɣ+**									
		mice		1	2	3	4	mean	s.d
Cytokine (pg/ml)	Cont-Patch	E:T	50:1	26.50	379.00	416.50	500.25	330.56	208.9
			100:1	-198.50	247.75	349.00	645.25	260.88	349.6
	OVA-Patch	E:T	50:1	862.75	970.25	1145.25	1290.25	1067.13	188.9
			100:1	854.00	981.50	1260.25	1579.00	1168.69	321.9

### Analysis of the dependency of the level of immunization on the amount of dissolved antigen

**Fig 7:**
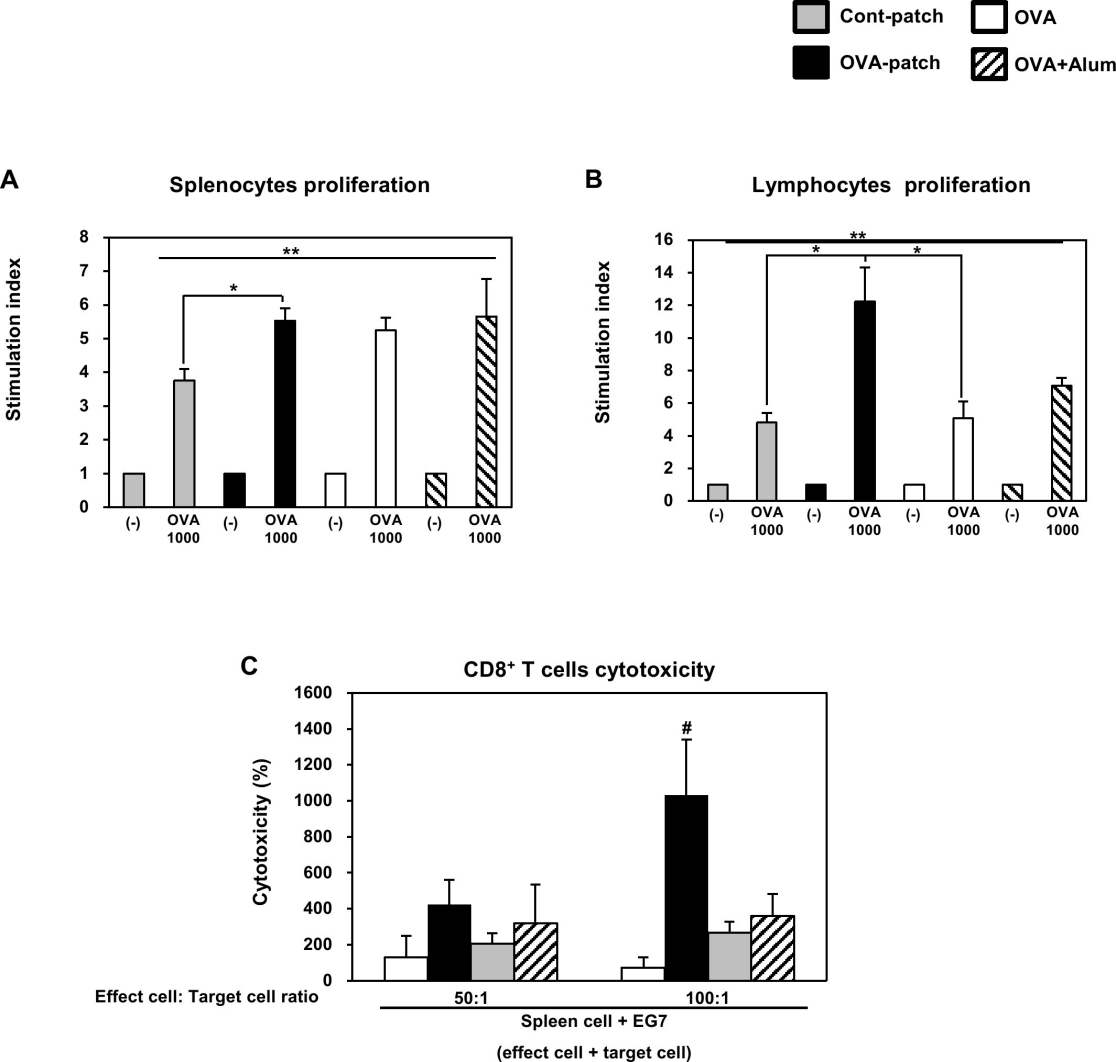
Efficiency of antigen-specific response depends on the immunization route. The mice (n = 4/group) were immunized either using the dissolving-microneedle-patch or dissolving-microneedle-patch loaded OVA. The splenocytes and lymphocytes of each group were re-stimulated with OVA 7 days after the last immunization. The proliferation of the (A)splenocytes and (B)lymphocytes was assessed using a Bromo-kit. (C) Immunization by dissolving-microneedle-patch loaded OVA induces OVA-specific CTL activity. Data are represented as mean ± SD of four mice per group. *p<0.05, **p<0.01 or **p<0.001 (-) vs. re-stimulation OVA. per group. ^#^p<0.05, ^##^ p<0.01 or ^###^ p<0.001 control-patch group vs. OVA-patch group. Digital Object Identifier: 10.6084/m9.figshare.7679696.

Given that the dissolving-microneedle-patch loaded OVA induces robust T cell responses in immunized mice, we decided to continue to inject OVA three times via the three different immunization injection routes to test these different routes of immunization, as compared with direct delivery to the skin. In this study, we injected OVA or OVA+Alum via intraperitoneal injection (i.p.) under the same conditions as those used for the dissolving-microneedle-patch loaded OVA. To determine the delivery of the dissolving-microneedle-patch loaded OVA and to test the level of immunization against OVA obtained using the two delivery route, ip injection and patch attachment, the splenocytes and lymphocytes from each experimental mouse were subjected to OVA re-stimulation. Likewise, to test the dependency of the level of immunization on the amount of dissolved antigen, OVA at concentrations of 1000 μg/ml was re-stimulated. As shown in Fig 7A and 7B, after OVA re-stimulation, the splenocytes and lymphocytes from the mice immunized with OVA exhibited an enhanced cell proliferation response. In the case of the splenocytes, the three different immunization injection routes showed the similar effects. However, the lymphocytes of the mice immunized by the dissolving-microneedle-patch loaded OVA proliferated to a greater extent than those of the mice immunized by the different immunization injection routes. To determine whether or not the antigen-specific CD8^+^ T cells were cytolytic, the splenocytes were co-incubated with EG7 cells. The mice immunized by dissolving-microneedle-patch loaded OVA exhibited significantly higher CTL activity than the mice immunized by OVA alone and OVA+Alum (Fig 7C). These results indicate that immunization by the dissolving-microneedle-patch loaded OVA enhanced the cell-mediated immune response in comparison to immunization by the antigen alone or antigen+Alum (Fig 7).

Therefore, a model antigen (OVA) was dissolved onto microneedle-patch to test the antigen release by conducting a transdermal delivery immunization study in mice.

It was found that immunization by means of the dissolving-microneedle-patch loaded OVA resulted in robust CD4^+^ T cells and CD8^+^ T cells responses comparable to those obtained after intraperitoneal injection immunization with OVA alone or OVA+Alum. In conclusion, antigen transdermal delivery by dissolving-microneedle-patch loaded antigens is more efficient than the ‘Intraperitoneal injection immunization’ approach, and in vivo vaccination studies show their applicability for the induction of both T cell responses. The values used to Fig 7A and 7B is Table 5. The values used to Fig 7C is Table 6.

**Table 5:** Efficiency of antigen-specific response depends on the immunization route by splenocytes proliferation, lymphocytes proliferation.

**Splenocytes Proliferation**									
		mice		1	2	3	4	mean	s.d
Stimulation Index	Cont-Patch	(-)		1.00	1.00	1.00	1.00	1.00	0.0
		re-stimulation OVA(μg/ml)	1000	4.07	3.64	4.00	3.32	3.76	0.3
	OVA-Patch	(-)		1.00	1.00	1.00	1.00	1.00	0.0
		re-stimulation OVA(μg/ml)	1000	5.45	6.05	5.42	5.20	5.53	0.4
	OVA	(-)		1.00	1.00	1.00	1.00	1.00	0.0
		re-stimulation OVA(μg/ml)	1000	5.50	5.36	4.70	5.45	5.25	0.4
	OVA+Alum	(-)		1.00	1.00	1.00	1.00	1.00	0.0
		re-stimulation OVA(μg/ml)	1000	4.83	5.09	5.40	7.29	5.65	1.1
**Lymphocytes Proliferation**									
		mice		1	2	3	4	mean	s.d
Stimulation Index	Cont-Patch	(-)		1.00	1.00	1.00	1.00	1.00	0.0
		re-stimulation OVA(μg/ml)	1000	4.56	4.78	5.65	4.32	4.83	0.6
	OVA-Patch	(-)		1.00	1.00	1.00	1.00	1.00	0.0
		re-stimulation OVA(μg/ml)	1000	14.96	12.59	11.31	10.05	12.23	2.1
	OVA	(-)		1.00	1.00	1.00	1.00	1.00	0.0
		re-stimulation OVA(μg/ml)	1000	4.08	5.05	6.52	4.64	5.07	1.0
	OVA+Alum	(-)		1.00	1.00	1.00	1.00	1.00	0.0
		re-stimulation OVA(μg/ml)	1000	6.78	6.57	7.68	7.21	7.06	0.5

**Table 6:** Efficiency of antigen-specific response depends on the immunization route by CD8^+^ T cell cytotoxicity.

CD8+ T cell Cytotoxicity									
		mice	1	2	3	4	mean	s.d
Cytotoxicity, %	Cont-Patch	E:T	50:1	116.24	69.13	204.70	125.28	128.84	56.2
			100:1	22.12	175.25	65.98	29.01	73.09	70.8
	OVA-Patch	E:T	50:1	804.05	352.09	53.50	474.63	421.07	310.6
			100:1	1367.86	1073.01	833.81	842.14	1029.21	251.5
	OVA	E:T	50:1	196.21	260.55	241.01	121.86	204.91	61.6
			100:1	211.25	350.02	270.24	238.02	267.38	60.1
	OVA+Alum	E:T	50:1	184.39	406.63	248.53	434.99	318.64	121.4
			100:1	301.85	297.49	410.29	428.85	359.62	69.7

In vivo anti-tumor therapeutic effect of grafted ovalbumin-expressing EG7 tumor cells in mice was delivered to the skin by dissolving-microneedle-patch loaded OVA

**Fig 8:**
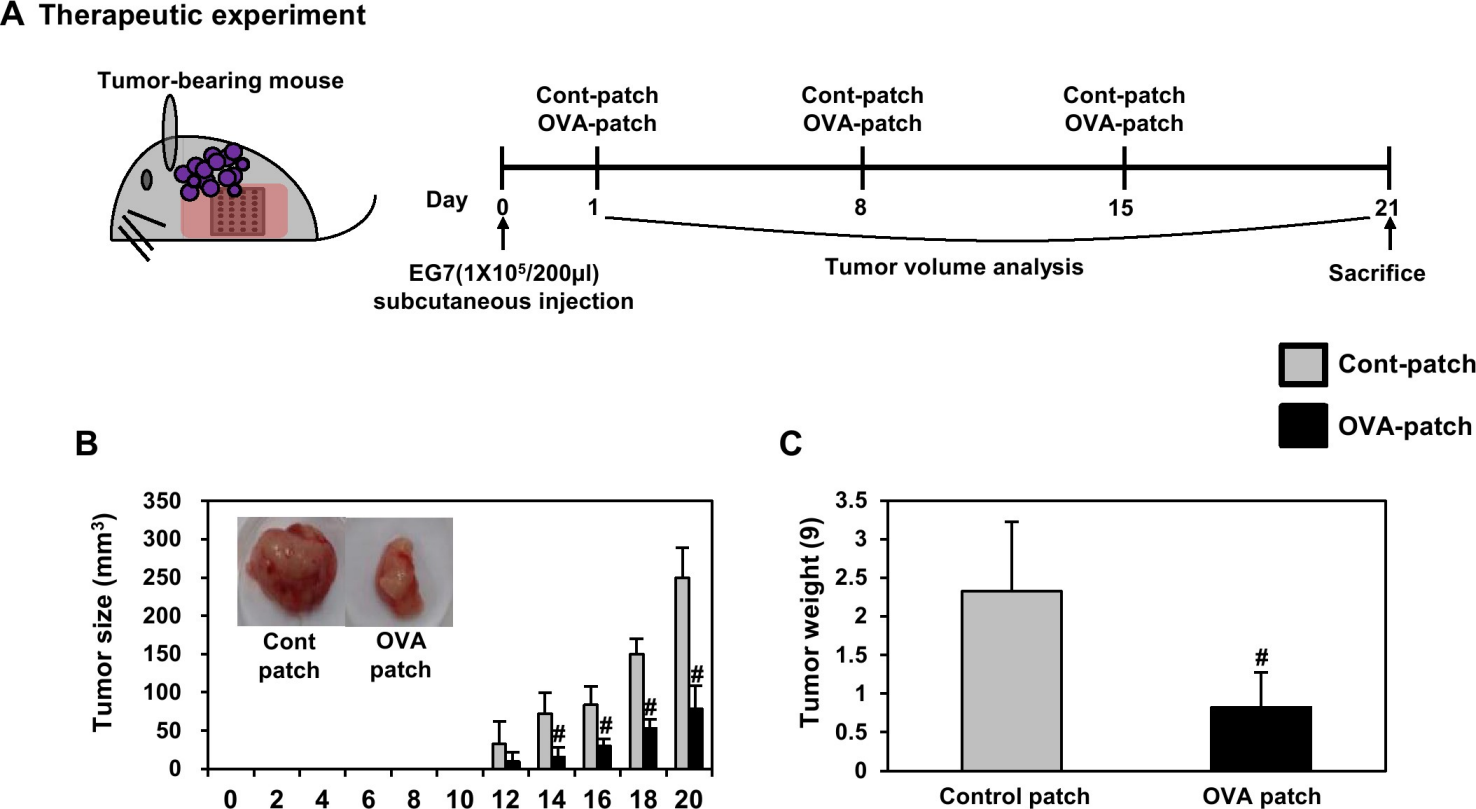
Analysis of anti-tumor immunity for therapeutic effect. 1 weeks after immunization by dissolving-microneedle-patch loaded OVA, 1×10^5^ EG7-OVA tumor cells were inoculated for tumor-challenge. (A) Experimental scheme for the anti-tumor immunity therapy (B) Tumor growth was monitored by measuring the tumor volume of mice. Photographs of the dissected tumor tissue from each group are also shown. (C) Tumor weight of the dissected tumor tissue from each group was also shown. Data are represented as mean ± SD of four mice per group. ^#^p<0.05, ^##^ p<0.01 or ^###^ p<0.001 control-patch group vs. OVA-patch group. Digital Object Identifier: 10.6084/m9.figshare.7679705.

Based on the previous results, we investigated the efficacy of the immunization on the suppression of grafted OVA-expressing EG7 tumor cells in mice. We also investigated whether the dissolving-microneedle-patch loaded OVA can be used as an antigen delivery system for cancer immunotherapy through in vivo analysis. To accomplish this, we attached the dissolving-microneedle-patch loaded OVA to the skin as a transdermal delivery vehicle into EG7-OVA tumor-bearing mice once a week for three weeks. The tumor volume was measured at 2-day intervals (n = 4). Both the dissolving-microneedle-patch loaded OVA led to significantly inhibited tumor growth (Fig 8B). Also, the inhibition of tumor weight exhibited a similar pattern (Fig 8C). These experimental results suggest that transdermal delivery systems using microneedle patches can deliver antigen and stimulated immune cells, thereby successfully inhibiting tumor proliferation and producing sufficient therapeutic effects.

We also evaluated the response of the stimulated immune cells ex vivo through transdermal delivery systems using the dissolving-microneedle-patch loaded OVA. Also, we next quantified the antigen-specific proliferation of splenocytes and lymphocytes in response to OVA protein stimulation to the delivery of protein antigen to verify their in vivo vaccination activity through transdermal delivery systems using the microneedle patch. The results of that, the maximum splenocytes proliferation in response to the dissolving-microneedle-patch loaded OVA was about 2.5 and 3.3 -fold higher than that in response to control group 100, 1000 μg/ml OVA re-stimulation (S1A Fig). The maximum IL-2 and, IFN-γ concentrations in the supernatant were also significantly higher on treatment with the dissolving-microneedle-patch loaded OVA compared with control group (S1A Fig and S1 Table). The proliferation of lymphocytes in response to dissolving-microneedle-patch loaded OVA was about 1.9 and 2.5 -fold strongly higher than that in response to control group 100, 1000 μg/ml OVA re-stimulation (S1B Fig and S1 Table). The maximum IL-2, IFN-γ concentration in the supernatant was also significantly higher on treatment with dissolving-microneedle-patch loaded OVA compared with treatment with control microneedle patch (S1B Fig and S1 Table). These data indicate that the transdermal delivery systems using the microneedle patch enhanced the cell-mediated immune response through improved antigen delivery. The values used to Fig 8B is Table 7. The values used to Fig 8C is Table 8.

**Table 7:** Analysis of tumor volume until day 20 by anti-tumor immunity for therapeutic effect.

**Tumor Size (mm**^**3**^**)**								
		mice	1	2	3	4	mean	s.d
**Tumor Size,** **mm**^**3**^	Cont-Patch	0 day	N.D.					
		2 day						
		4 day						
		6 day						
		8 day						
		10 day						
		12 day	0.00	70.71	33.76	24.90	32.34	29.3
		14 day	62.49	92.31	91.92	42.89	72.40	26.7
		16 day	77.05	85.05	118.13	53.63	83.46	24.1
		18 day	142.48	123.22	167.21	164.77	149.42	20.7
		20 day	293.99	227.88	269.14	207.67	249.67	39.1
	OVA-Patch	0 day	N.D.					
		2 day						
		4 day						
		6 day						
		8 day						
		10 day						
		12 day	0.00	25.84	11.74	0.00	9.40	12.3
		14 day	0.00	27.69	21.75	12.99	15.61	12.0
		16 day	17.12	40.22	27.25	33.61	29.55	9.8
		18 day	36.41	60.02	61.05	55.00	53.12	11.4
		20 day	35.70	82.87	105.01	91.42	78.75	30.1

N.D.: Not Detection

**Table 8:** Analysis of tumor weight by anti-tumor immunity for therapeutic effect.

Tumor Weight (g)								
		mice	1	2	3	4	mean	s.d
Tumor Weight, (g)	Cont-Patch		2.25	2.14	3.55	1.38	2.33	0.9
	OVA-Patch		0.20	0.92	1.29	0.89	0.82	0.5

### In vivo anti-tumor prophylactic effect of grafted OVA-expressing EG7 tumor cells in mice was delivered to the skin by the dissolving-microneedle-patch loaded OVA

**Fig 9:**
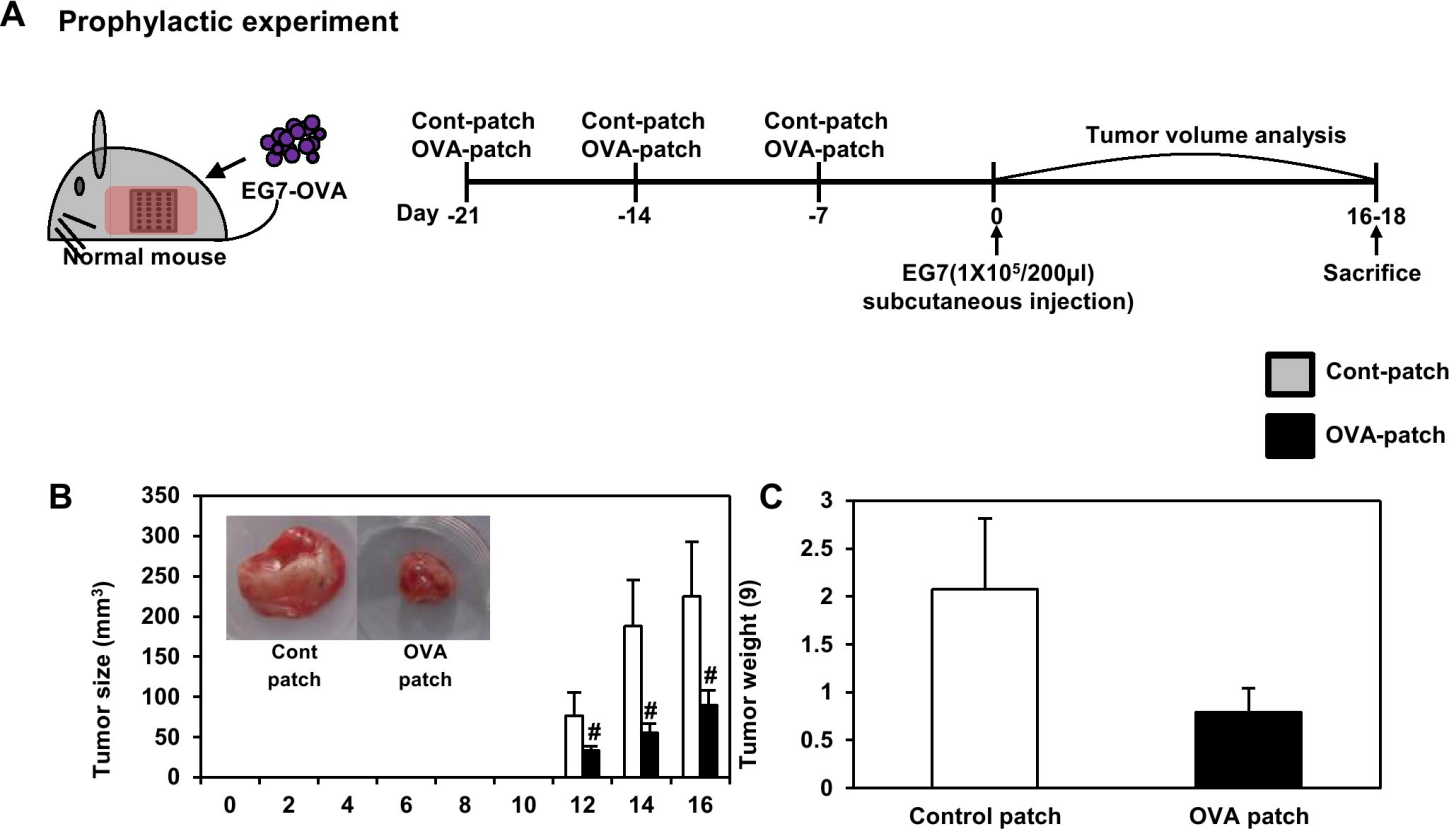
Analysis of anti-tumor immunity for prophylactic effect. EG7-OVA tumor cells (1×10^5^ cells) were inoculated subcutaneously into mice (n = 4/group) 1days before immunization by dissolving-microneedle-patch loaded OVA. (A) Experimental scheme for the anti-tumor immunity therapy (B) Tumor growth was monitored by measuring the tumor volume of mice. Photographs of the dissected tumor tissue from each group are also shown. (C) Tumor weight of the dissected tumor tissue from each group was also shown. Data are represented as mean ± SD of four mice per group. ^#^p<0.05, ^##^ p<0.01 or ^###^ p<0.001 control-patch group vs. OVA-patch group. Digital Object Identifier: 10.6084/m9.figshare.7679711.

We also investigated whether the transdermal delivery systems using the microneedle patch can be used as a prophylactic vaccine for tumor immunotherapy through in vivo analysis. Although most tumor immunotherapies have focused on the use of therapeutic immune cells or drugs after a tumor was detected, adjuvant systems designed to induce a prophylactic anti-tumor immune response that can boost the body’s immunity, in order to prevent tumor recurrence and growth, could be an alternative strategy for tumor control. In the same manner, OVA was selected as a tumor antigen, and the EG7-OVA tumor model was used for the in vivo experiments. Inspired by the promising preliminary results on the use of the dissolving-microneedle-patch loaded OVA as an immune-stimulating antigen delivery system for the induction of anti-tumor immunity, an in vivo tumor challenge trial was conducted. One week after the final immunization, tumor cells were inoculated. The tumor volume was measured at 2-day intervals (n = 4). Both dissolving-microneedle-patch loaded OVA groups showed significantly inhibited tumor growth (Fig 9B) and tumor weight (Fig 9C). In the same manner, We evaluated the response of the stimulated immune cells ex vivo through the transdermal delivery system using the dissolving-microneedle-patch. The proliferation of splenocytes in response to the dissolving-microneedle-patch loaded OVA was about 2.1, and 2.6 -fold higher than that in response to control group at 100, 1000 μg/ml OVA re-stimulation (S2A Fig and S2 Table). The maximum IL-2, and IFN-γ concentrations in the supernatant were also significantly higher on treatment with the dissolving-microneedle-patch loaded OVA compared with group of control-microneedle patch. The maximum lymphocytes proliferation in response to the dissolving-microneedle-patch loaded OVA was about 1.4, and 1.9 -fold higher than that in response to control group 100, 1000 μg/ml OVA re-stimulation (S2B Fig and S2 Table). The maximum IL-2, and IFN-γ concentrations in the supernatant was also significantly higher on treatment with dissolving-microneedle-patch loaded OVA compared with control group (S2B Fig and S2 Table). These experimental results suggest that the patch can potently deliver the antigen and thereby enhance the cellular immune response afforded by the T cell response. The values used to Fig 9B is Table 9. The values used to Fig 9C is Table 10.

**Table 9:** Analysis of tumor volume until day 16 by anti-tumor immunity for prophylactic effect.

Tumor Size (mm^3^)								
		mice	1	2	3	4	mean	s.d
**Tumor Size,** **mm**^**3**^	Cont-Patch	0 day	N.D.					
		2 day						
		4 day						
		6 day						
		8 day						
		10 day						
		12 day	156.37	579.89	134.70	279.44	287.60	177.6
		14 day	647.31	1749.33	916.03	1034.88	1086.89	470.4
		16 day	750.07	2164.05	1542.39	1076.21	1383.18	613.8
	OVA-Patch	0 day	N.D.					
		2 day						
		4 day						
		6 day						
		8 day						
		10 day						
		12 day	97.12	79.97	117.21	61.23	88.88	23.9
		14 day	176.97	172.71	230.08	124.73	176.12	43.1
		16 day	251.90	441.67	464.11	262.35	355.01	113.5

N.D.: Not Detection

**Table 10:** Analysis of tumor weight by anti-tumor immunity for prophylactic effect.

Tumor Weight (g)								
		mice	1	2	3	4	mean	s.d
**Tumor** **Weight (g)**	Cont-Patch		2.13	1.02	2.70	2.45	2.07	0.7
	OVA-Patch		0.95	0.53	0.63	1.06	0.79	0.3

Collectively, these experimental results suggest that antigen delivery systems using the dissolving-microneedle-patch loaded OVA can elicit potent prophylactic anti-cancer immunity, as well as a therapeutic response.

## Discussion

A strategically important target of vaccination is the induction of a specific immune response through the interaction of Th1 cells and cytotoxic T cells. Also, vaccination based on a strong cellular response is needed to induce an immune response against the tumor and chronic infection [31]. Accordingly, effective vaccination should allow a specific cellular response to be induced, enhanced and maintained against tumor and chronic infection [31]. The skin is a strong target for vaccine delivery systems because the epidermis and dermis of the skin contain a high density of antigen presenting cells capable of capturing and presenting vaccine antigens [32–34]. Overall, the microneedle patch is a very versatile delivery technology, allowing for the easy and reproducible delivery of antigens to the skin for efficient vaccination [1]. Vaccines delivered to the skin through the microneedle of the antigen delivery system should directly transfer the antigen into the epidermal and dermal compartments, because of the presence of professional antigen-presenting cells [1,35]. Transcutaneously administered vaccine antigens have to induce an immune response once they are delivered into the skin, captured by antigen presenting cells and reach the lymph nodes [36]. DCs are the most potent and professional antigen presenting cells and play a pivotal role in adaptive immunity as initiators and modulators of T cell immune responses [15,31]. Immature DCs activate T cells weakly and constantly engulf and process soluble and particulate antigens through various mechanisms and then begin to mature and migrate to the lymph nodes [15,31]. During their maturation, the DCs present the processed protein antigens in the form of linear peptide epitopes to CD4^+^ and CD8^+^ T cells through major histocompatibility complex (MHC) class I and class II molecules to initiate a proper adaptive immune response against the antigens [15,31]. The Th1 cell immune response through CD4^+^ T cells is very important in establishing cellular immunity against pathogens and tumors [23–25]. Also, CD8^+^ T cells have been shown to be potent mediators of anti-tumor immunity [5,36–38]. In the case of CD8^+^ T cells, tumor-directed immune-based therapies have focused on eliciting a CTL response, so that cytotoxic T cells can directly kill tumors [5,36–38]. In addition, CD8^+^ T cells are key cellular components in the control of many intracellular infections, many putative tumor antigens are intracellular proteins, and CTLs respond to the peptides present in MHC class I molecules, which are most often derived from intracellular proteins [5,36,37]. The CD4^+^ T cell and CD8^+^ T cell responses though antigen-specific can help broaden the immune response for the development of a universal vaccine [5,36–39]. To demonstrate this process, we immunized the mice transcutaneously with an dissolving-microneedle-patch loaded antigens [1,5,15,32,39]. Then, we focused on the induction or enhancement of the cellular immune response against the target of interest based on its antigen-specificity. After the 3rd boosting immunization, the endogenous OVA-specific immune response was evaluated by determining the enhanced cell proliferation induced by the splenocytes and lymphocytes (Fig 4). Also, after OVA re-stimulation, the cytokine levels in the splenocytes and lymphocytes obtained from the mice immunized with the dissolving-microneedle-patch loaded OVA were found to secrete considerably more cytokines (IL-2, and IFN-γ) than the control mice (Fig 4). Also, the antigen-specific immune response from the antigens delivered via the patch was investigated to determine whether a significantly enhanced Th1 cell and cytotoxic T cell (CD4^+^ T cells and CD8^+^ T cells) response can be achieved. As shown in Fig 5, we confirmed that IFN-γ secretions through these cells were enhanced when re-stimulated with OVA. These results demonstrated that the OVA within the dissolving-microneedle-patch loaded and delivered the OVA antigen and induced an antigen-specific Th1 cells and cytotoxic T cells mediated immune response. The results shown in Fig 6 suggest that the dissolving-microneedle-patch loaded OVA induced an OVA-specific CTL response that targeted the EG7 tumor cells through the cytolytic activity of the responding T cells, and this process was tested directly by measuring the OVA-expressing EG7 target cells using the LDH-release assay and the induction of OVA-expressing EG7-specific IFN-γ-producing CD8^+^ cells. Collectively, these results indicate that when the dissolving-microneedle-patch loaded OVA protein as an antigen is delivered to the skin, the OVA released will induce an immune response and, then, the OVA-specific CTLs will have the potential to kill the OVA-expressing tumor cells in vivo. Accordingly, the present results show that microneedle-based immunization is highly suitable for effectively inducing an OVA-specific CD8^+^ T cell response.

Based on these comprehensive results, we confirmed that transdermal delivery systems using the dissolving-microneedle-patch loaded OVA can be used as a therapeutic or prophylactic vaccine for tumor immunotherapy. The result was very interesting. Through tumor challenge experiments, the mice immunized with these transdermal delivery systems exhibited significant CTL-mediated enhanced cytotoxicity, antigen-specific response, and IFN-γ secretion. Finally, we confirmed that these transdermal delivery systems contributed to the inhibition of tumor growth and prevention of tumor formation in the context of the therapeutic and prophylactic vaccine, respectively (Figs 8 and 9).

Our study shows that the dissolving-microneedle-patch is a very versatile delivery technology, allowing easier antigen delivery to the skin for efficient vaccination compared with vaccination by injection. Also, the advantage of these patch is that it has the advantage of delivering the antigen to the dendritic cells in the subcutaneous space without pain, easily and conveniently with a long needle pointed, more than other patches [11]. However, the disadvantage of our patch is that it must be attached for 3h.

In this study, these delivery systems enhanced the anti-tumor activity through the Th1 cells and cytotoxic T cells mediated immune response. These delivery systems enhanced the anti-tumor activity through the Th1 cells and cytotoxic T cells mediated immune response. In conclusion, we are suggest that transdermal delivery systems using a dissolving-microneedle-patch have god potential to be used as efficient vaccination systems for the purpose of inducing an anti-tumor immune response.

## Conclusions

In summary, our study demonstrated the applicability of dissolving-microneedle-patch loaded antigens for vaccination purposes by using OVA as a model antigen for the induction of an immune response, as well as the inhibition of tumor growth and prevention of tumor formation in the context of the therapeutic and prophylactic vaccines, respectively, through the effectively increased OVA-specific CD4^+^/CD8^+^ T cell response and induced OVA-specific CTL response against the graft of OVA-expressing EG7 tumor cells in the immunized mice. These data show the potential of the dissolving-microneedle-patch to act as an attractive antigen delivery vehicle.

## Supporting information

S1 Fig Analysis of anti-tumor immunity for therapeutic effect. Digital Object Identifier: 10.6084/m9.figshare.7679723.
(TIF)

S2 Fig Analysis of anti-tumor immunity for prophylactic effect. Digital Object Identifier: 10.6084/m9.figshare.7679729.
(TIF)

S1 Table Analysis of anti-tumor immunity for therapeutic effect. (DOCX)

S2 Table Analysis of anti-tumor immunity for prophylactic effect. (DOCX)
