# Supplementary figures and images for: Enhanced anti-tumor immunotherapy by dissolving microneedle patch loaded ovalbumin
Source: PLoS One. 2019 Aug 6;14(8):e0220382. doi: 10.1371/journal.pone.0220382 (PMC6684091; doi:10.1371/journal.pone.0220382)

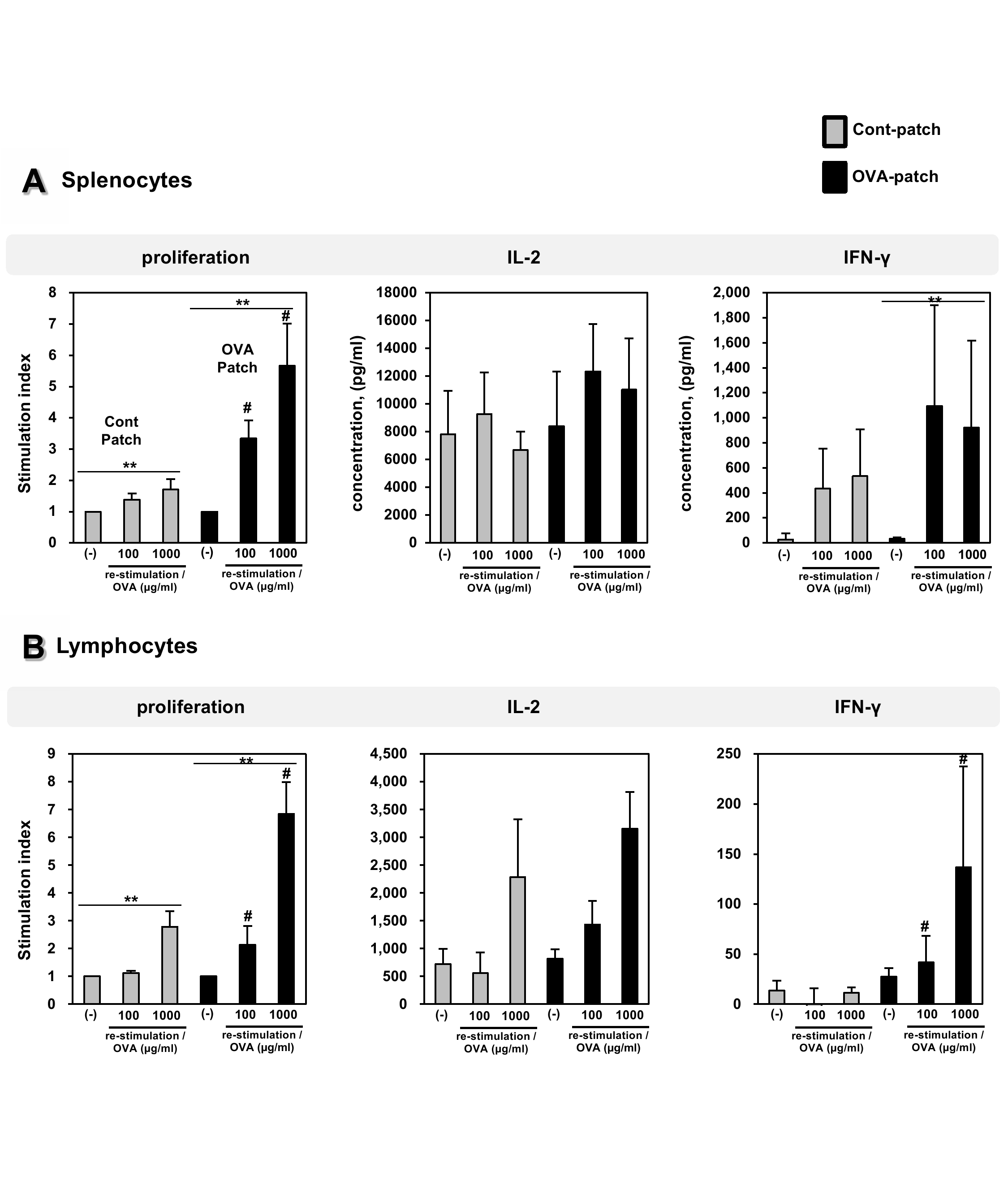

Supplement: S1 Fig — Digital Object Identifier: 10.6084/m9.figshare.7679723. (TIF) [file pone.0220382.s001.tif]

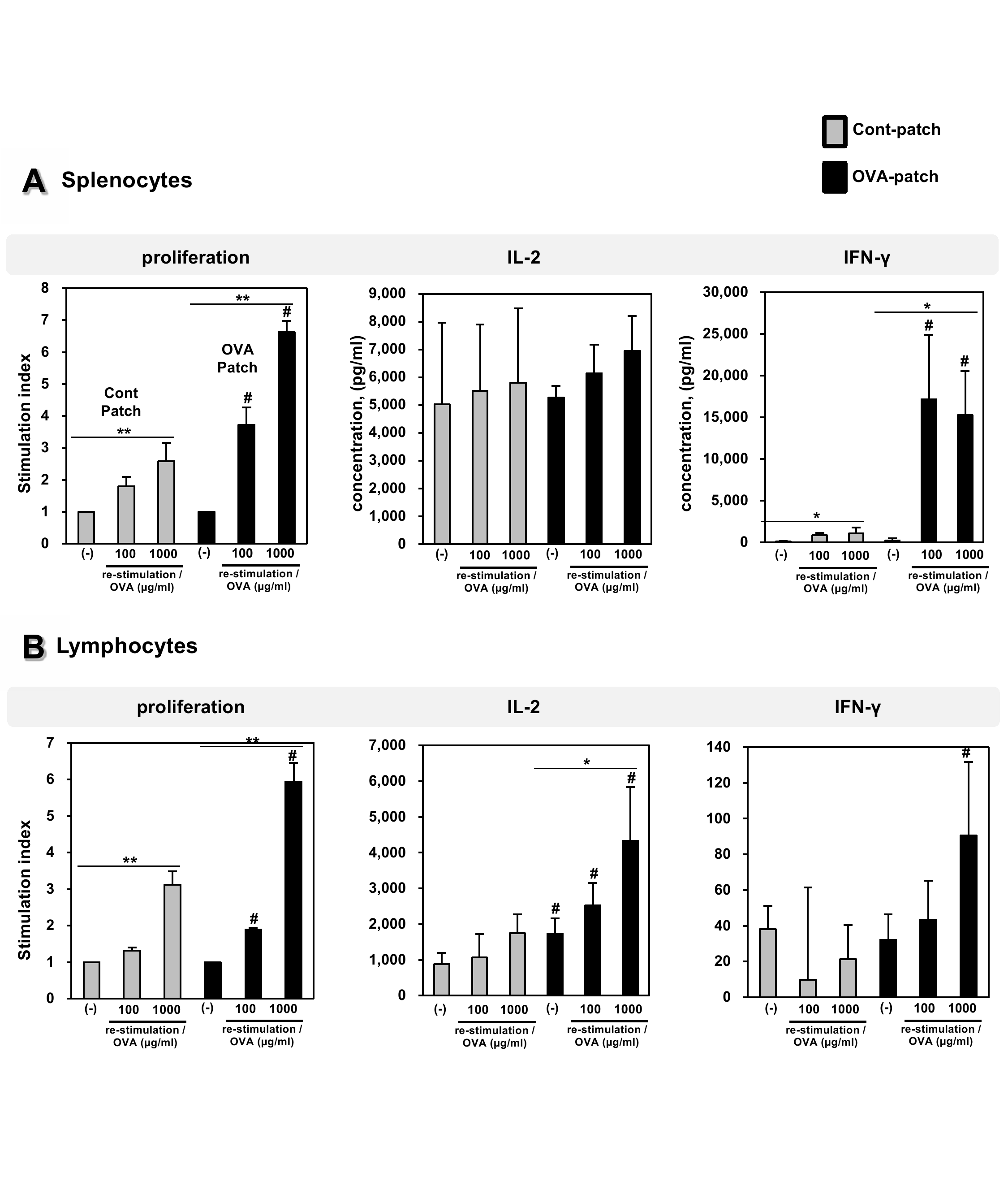

Supplement: S2 Fig — Digital Object Identifier: 10.6084/m9.figshare.7679729. (TIF) [file pone.0220382.s002.tif]
