# Supplementary material for: Enhanced anti-tumor immunotherapy by dissolving microneedle patch loaded ovalbumin
Source: PLoS One. 2019 Aug 6;14(8):e0220382. doi: 10.1371/journal.pone.0220382 (PMC6684091; doi:10.1371/journal.pone.0220382)
Supplement: S1 Table — (DOCX) [file pone.0220382.s003.docx]

**S1 Table. Analysis of anti-tumor immunity for therapeutic effect.**

| **Splenocytes Proliferation** | | | | | | | | | |  |  |  |
| --- | --- | --- | --- | --- | --- | --- | --- | --- | --- | --- | --- | --- |
|  |  | mice | | 1 | 2 | 3 | 4 | mean | s.d |  |  |  |
| Stimulation Index | Cont-Patch | (-) | | 1.00 | 1.00 | 1.00 | 1.00 | 1.00 | 0.0 |  |  |  |
|  |  | re-stimulation  OVA(μg/ml) | 100 | 1.51 | 1.32 | 1.28 | 1.38 | 1.38 | 0.2 |  |  |  |
|  |  |  | 1000 | 2.00 | 1.52 | 1.56 | 1.74 | 1.70 | 0.3 |  |  |  |
|  | OVA-Patch | (-) | | 1.00 | 1.00 | 1.00 | 1.00 | 1.00 | 0.0 |  |  |  |
|  |  | re-stimulation  OVA(μg/ml) | 100 | 3.73 | 2.93 | 3.22 | 3.48 | 3.34 | 0.6 |  |  |  |
|  |  |  | 1000 | 6.69 | 4.51 | 5.94 | 5.52 | 5.66 | 1.4 |  |  |  |
| **Splenocytes IL-2** | | | | | | | | | |  |  |  |
|  |  | mice | | 1 | 2 | 3 | 4 | mean | s.d |  |  |  |
| Cytokine  (pg/ml) | Cont-Patch | (-) | | 11118.50 | 8588.50 | 3583.50 | 7988.50 | 7819.75 | 3133.0 |  | |  |
|  |  | re-stimulation  OVA(μg/ml) | 100 | 12311.00 | 10796.00 | 5423.50 | 8543.50 | 9268.50 | 2994.4 |  |  |  |
|  |  |  | 1000 | 8473.50 | 5371.00 | 6186.00 | 6763.50 | 6698.50 | 1314.0 |  |  |  |
|  | OVA-Patch | (-) | | 12536.00 | 8413.50 | 9556.00 | 3121.00 | 8406.63 | 3929.0 |  | |  |
|  |  | re-stimulation  OVA(μg/ml) | 100 | 16808.50 | 11553.50 | 12418.50 | 8581.00 | 12340.38 | 3402.1 |  |  |  |
|  |  |  | 1000 | 15293.50 | 8976.00 | 12741.00 | 7123.50 | 11033.50 | 3678.1 |  |  |  |
| **Splenocytes IFN-Ɣ** | | | | | | | | | |  |  |  |
|  |  | mice | | 1 | 2 | 3 | 4 | mean | s.d |  |  |  |
| Cytokine  (pg/ml) | Cont-Patch | (-) | | 31.24 | 93.00 | 10.65 | -24.94 | 27.49 | 49.5 |  | |  |
|  |  | re-stimulation  OVA(μg/ml) | 100 | 753.59 | 481.53 | 512.12 | -14.06 | 433.29 | 322.1 |  |  |  |
|  |  |  | 1000 | 937.41 | 688.59 | 452.12 | 56.53 | 533.66 | 374.8 |  |  |  |
|  | OVA-Patch | (-) | | 30.94 | 22.12 | 25.94 | 48.29 | 31.82 | 11.6 |  | |  |
|  |  | re-stimulation  OVA(μg/ml) | 100 | 129.76 | 1315.65 | 879.47 | 2054.47 | 1094.84 | 805.7 |  |  |  |
|  |  |  | 1000 | 145.94 | 853.88 | 844.47 | 1839.47 | 920.94 | 696.3 |  |  |  |
| **Lymphocytes Proliferation** | | | | | | | | | |  |  |  |
|  |  | mice | | 1 | 2 | 3 | 4 | mean | s.d |  |  |  |
| Stimulation Index | Cont-Patch | (-) | | 1.00 | 1.00 | 1.00 | 1.00 | 1.00 | 0.0 |  |  |  |
|  |  | re-stimulation  OVA(μg/ml) | 100 | 1.09 | 1.23 | 1.12 | 1.02 | 1.12 | 0.1 |  |  |  |
|  |  |  | 1000 | 2.02 | 3.06 | 3.30 | 2.76 | 2.79 | 0.6 |  |  |  |
|  | OVA-Patch | (-) | | 1.00 | 1.00 | 1.00 | 1.00 | 1.00 | 0.0 |  |  |  |
|  |  | re-stimulation  OVA(μg/ml) | 100 | 2.96 | 2.39 | 1.48 | 1.70 | 2.13 | 0.7 |  |  |  |
|  |  |  | 1000 | 6.96 | 8.39 | 5.76 | 6.28 | 6.85 | 1.1 |  |  |  |
| **Lymphocytes IL-2** | | | | | | | | | |  |  |  |
|  |  | mice | | 1 | 2 | 3 | 4 | mean | s.d |  |  |  |
| Cytokine  (pg/ml) | Cont-Patch | (-) | | 486.00 | 608.50 | 683.50 | 1108.50 | 721.63 | 270.5 |  | | |
|  |  | re-stimulation  OVA(μg/ml) | 100 | 1108.50 | 446.00 | 318.50 | 353.50 | 556.63 | 371.8 |  |  |  |
|  |  |  | 1000 | 2966.00 | 748.50 | 2573.50 | 2866.00 | 2288.50 | 1040.1 |  |  |  |
|  | OVA-Patch | (-) | | 993.50 | 728.50 | 701.00 | 863.50 | 821.63 | 161.5 |  | | |
|  |  | re-stimulation  OVA(μg/ml) | 100 | 1703.50 | 856.00 | 1193.50 | 1981.00 | 1433.50 | 426.7 |  |  |  |
|  |  |  | 1000 | 3041.00 | 2221.00 | 3518.50 | 3851.00 | 3157.88 | 656.2 |  |  |  |
| **Lymphocytes IFN-Ɣ** | | | | | | | | | |  |  |  |
|  |  | mice | | 1 | 2 | 3 | 4 | mean | s.d |  |  |  |
| Cytokine  (pg/ml) | Cont-Patch | (-) | | 9.47 | 4.18 | 27.12 | 13.29 | 13.51 | 9.8 |  | | |
|  |  | re-stimulation  OVA(μg/ml) | 100 | 14.76 | 8.29 | -42.88 | -42.29 | -15.53 | 31.4 |  |  |  |
|  |  |  | 1000 | 12.71 | 17.41 | 10.94 | 4.18 | 11.31 | 5.5 |  |  |  |
|  | OVA-Patch | (-) | | 40.06 | 23.00 | 22.12 | 24.18 | 27.34 | 8.5 |  | | |
|  |  | re-stimulation  OVA(μg/ml) | 100 | 18.88 | 19.76 | 55.94 | 72.12 | 41.68 | 26.6 |  |  |  |
|  |  |  | 1000 | 59.76 | 61.24 | 153.88 | 272.71 | 136.90 | 100.7 |  |  |  |
