# Supplementary material for: Enhanced anti-tumor immunotherapy by dissolving microneedle patch loaded ovalbumin
Source: PLoS One. 2019 Aug 6;14(8):e0220382. doi: 10.1371/journal.pone.0220382 (PMC6684091; doi:10.1371/journal.pone.0220382)
Supplement: S2 Table — (DOCX) [file pone.0220382.s004.docx]

**S2 Table. Analysis of anti-tumor immunity for prophylactic effect.**

| **Splenocytes Proliferation** | | | | | | | | | |
| --- | --- | --- | --- | --- | --- | --- | --- | --- | --- |
|  |  | mice | | 1 | 2 | 3 | 4 | mean | s.d |
| Stimulation Index | Cont-Patch | (-) | | 1.00 | 1.00 | 1.00 | 1.00 | 1.00 | 0.0 |
|  |  | re-stimulation  OVA(μg/ml) | 100 | 1.81 | 2.17 | 1.77 | 1.43 | 1.80 | 0.3 |
|  |  |  | 1000 | 2.86 | 3.25 | 2.30 | 1.94 | 2.59 | 0.6 |
|  | OVA-Patch | (-) | | 1.00 | 1.00 | 1.00 | 1.00 | 1.00 | 0.0 |
|  |  | re-stimulation  OVA(μg/ml) | 100 | 2.95 | 3.77 | 4.18 | 4.01 | 3.73 | 0.5 |
|  |  |  | 1000 | 6.23 | 7.07 | 6.72 | 6.46 | 6.62 | 0.4 |
| **Splenocytes IL-2** | | | | | | | | | |
|  |  | mice | | 1 | 2 | 3 | 4 | mean | s.d |
| Cytokine  (pg/ml) | Cont-Patch | (-) | | 2105.00 | 6610.00 | 3065.00 | 8325.00 | 5026.25 | 2930.9 |
|  |  | re-stimulation  OVA(μg/ml) | 100 | 2420.00 | 7070.00 | 4890.00 | 7685.00 | 5516.25 | 2387.2 |
|  |  |  | 1000 | 3020.00 | 7500.00 | 4110.00 | 8600.00 | 5807.50 | 2665.5 |
|  | OVA-Patch | (-) | | 5760.00 | 5425.00 | 5100.00 | 4805.00 | 5272.50 | 412.0 |
|  |  | re-stimulation  OVA(μg/ml) | 100 | 6050.00 | 7240.00 | 6495.00 | 4800.00 | 6146.25 | 1023.0 |
|  |  |  | 1000 | 6300.00 | 8245.00 | 7720.00 | 5520.00 | 6946.25 | 1256.6 |
| **Splenocytes IFN-Ɣ** | | | | | | | | | |
|  |  | mice | | 1 | 2 | 3 | 4 | mean | s.d |
| Cytokine  (pg/ml) | Cont-Patch | (-) | | 130.78 | 174.67 | 110.78 | 106.89 | 130.78 | 31.1 |
|  |  | re-stimulation  OVA(μg/ml) | 100 | 844.11 | 1213.56 | 596.33 | 725.78 | 844.94 | 265.8 |
|  |  |  | 1000 | 972.44 | 2076.33 | 370.78 | 863.00 | 1070.64 | 719.7 |
|  | OVA-Patch | (-) | | 23.00 | 632.44 | 70.78 | 213.00 | 234.81 | 277.1 |
|  |  | re-stimulation  OVA(μg/ml) | 100 | 5823.56 | 18936.89 | 22233.00 | 21745.78 | 17184.81 | 7712.2 |
|  |  |  | 1000 | 7672.44 | 17206.89 | 16652.44 | 19626.89 | 15289.67 | 5239.8 |
| **Lymphocytes Proliferation** | | | | | | | | | |
|  |  | mice | | 1 | 2 | 3 | 4 | mean | s.d |
| Stimulation Index | Cont-Patch | (-) | | 1.00 | 1.00 | 1.00 | 1.00 | 1.00 | 0.0 |
|  |  | re-stimulation  OVA(μg/ml) | 100 | 1.26 | 1.25 | 1.30 | 1.43 | 1.31 | 0.1 |
|  |  |  | 1000 | 2.81 | 3.38 | 2.79 | 3.49 | 3.12 | 0.4 |
|  | OVA-Patch | (-) | | 1.00 | 1.00 | 1.00 | 1.00 | 1.00 | 0.0 |
|  |  | re-stimulation  OVA(μg/ml) | 100 | 1.91 | 1.86 | 1.96 | 1.85 | 1.90 | 0.0 |
|  |  |  | 1000 | 5.32 | 6.11 | 6.53 | 5.82 | 5.95 | 0.5 |
| **Lymphocytes IL-2** | | | | | | | | | |
|  |  | mice | | 1 | 2 | 3 | 4 | mean | s.d |
| Cytokine  (pg/ml) | Cont-Patch | (-) | | 436.00 | 1126.00 | 846.00 | 1101.00 | 877.25 | 320.2 |
|  |  | re-stimulation  OVA(μg/ml) | 100 | 76.00 | 1431.00 | 1396.00 | 1351.00 | 1063.50 | 659.1 |
|  |  |  | 1000 | 1401.00 | 2161.00 | 1171.00 | 2236.00 | 1742.25 | 536.0 |
|  | OVA-Patch | (-) | | 1711.00 | 1781.00 | 2246.00 | 1211.00 | 1737.25 | 423.6 |
|  |  | re-stimulation  OVA(μg/ml) | 100 | 1641.00 | 3146.00 | 2676.00 | 2621.00 | 2521.00 | 632.2 |
|  |  |  | 1000 | 3101.00 | 6376.00 | 4566.00 | 3296.00 | 4334.75 | 1507.9 |
| **Lymphocytes IFN-Ɣ** | | | | | | | | | |
|  |  | mice | | 1 | 2 | 3 | 4 | mean | s.d |
| Cytokine  (pg/ml) | Cont-Patch | (-) | | 33.00 | 51.33 | 22.44 | 45.78 | 38.14 | 13.0 |
|  |  | re-stimulation  OVA(μg/ml) | 100 | -61.44 | 60.22 | 28.56 | 11.89 | 9.81 | 51.6 |
|  |  |  | 1000 | 47.44 | 23.00 | 3.56 | 11.33 | 21.33 | 19.2 |
|  | OVA-Patch | (-) | | 25.22 | 34.11 | 51.33 | 18.00 | 32.17 | 14.4 |
|  |  | re-stimulation  OVA(μg/ml) | 100 | 28.00 | 56.33 | 67.44 | 21.89 | 43.42 | 21.9 |
|  |  |  | 1000 | 65.22 | 136.33 | 113.00 | 47.44 | 90.50 | 41.2 |
